# A Membrane-Type-1 Matrix Metalloproteinase (MT1-MMP) – Discoidin Domain Receptor 1 Axis Regulates Collagen-Induced Apoptosis in Breast Cancer Cells

**DOI:** 10.1371/journal.pone.0116006

**Published:** 2015-03-16

**Authors:** Delphine Assent, Isabelle Bourgot, Benoît Hennuy, Pierre Geurts, Agnès Noël, Jean-Michel Foidart, Erik Maquoi

**Affiliations:** 1 Laboratory of Tumour and Developmental Biology, Groupe Interdisciplinaire de Génoprotéomique Appliqué (GIGA), Unit of Cancer, University of Liège, Liège, Belgium; 2 GIGA-Genomics platform, University of Liège, Liège, Belgium; 3 Systems and Modelling, Department of Electrical Engineering and Computer Science & GIGA-R, University of Liège, Liège, Belgium; Stony Brook University, UNITED STATES

## Abstract

During tumour dissemination, invading breast carcinoma cells become confronted with a reactive stroma, a type I collagen-rich environment endowed with anti-proliferative and pro-apoptotic properties. To develop metastatic capabilities, tumour cells must acquire the capacity to cope with this novel microenvironment. How cells interact with and respond to their microenvironment during cancer dissemination remains poorly understood. To address the impact of type I collagen on the fate of tumour cells, human breast carcinoma MCF-7 cells were cultured within three-dimensional type I collagen gels (3D COL1). Using this experimental model, we have previously demonstrated that membrane type-1 matrix metalloproteinase (MT1-MMP), a proteinase overexpressed in many aggressive tumours, promotes tumour progression by circumventing the collagen-induced up-regulation of BIK, a pro-apoptotic tumour suppressor, and hence apoptosis. Here we performed a transcriptomic analysis to decipher the molecular mechanisms regulating 3D COL1-induced apoptosis in human breast cancer cells. Control and MT1-MMP expressing MCF-7 cells were cultured on two-dimensional plastic plates or within 3D COL1 and a global transcriptional time-course analysis was performed. Shifting the cells from plastic plates to 3D COL1 activated a complex reprogramming of genes implicated in various biological processes. Bioinformatic analysis revealed a 3D COL1-mediated alteration of key cellular functions including apoptosis, cell proliferation, RNA processing and cytoskeleton remodelling. By using a panel of pharmacological inhibitors, we identified discoidin domain receptor 1 (DDR1), a receptor tyrosine kinase specifically activated by collagen, as the initiator of 3D COL1-induced apoptosis. Our data support the concept that MT1-MMP contributes to the inactivation of the DDR1-BIK signalling axis through the cleavage of collagen fibres and/or the alteration of DDR1 receptor signalling unit, without triggering a drastic remodelling of the transcriptome of MCF-7 cells.

## Introduction

Cells in multicellular organisms are surrounded by a complex three-dimensional (3D) macromolecular extracellular matrix (ECM). This matrix, traditionally thought to serve a structural function providing support and strength to cells within tissues, is increasingly being recognized as having pleiotropic effects in development and growth. Cell–matrix interactions play a central role in wound healing, developmental morphogenesis, and cancer metastasis. The ECM constitutes the physical microenvironment for cell anchorage and serves as a tissue scaffold, guides cell migration during embryonic development and wound repair, and has key roles in tissue morphogenesis. However, beyond these obvious scaffolding functions, the ECM is also responsible for transmitting environmental signals to cells, which affect essentially all aspects of a cell’s life, including its proliferation, differentiation and death [1]. Indeed, tissues are endowed with embedded regulatory programs for controlling aberrant proliferation of resident cells, as well as for inhibiting invasion of foreign cell types, which act by inducing cell death [2]. Although basic mechanisms implicated in cell-ECM interactions on 2D substrata are generally well understood, much less is known regarding these interactions in more physiological 3D matrix [3–5]. Understanding the mechanics of cancer cell behaviour in 3D microenvironments is therefore of paramount importance.

During metastasis, tumour cells encounter various ECM microenvironments with distinct composition and architecture, including basement membrane and interstitial collagen networks [6–8]. The interactions between cancer cells and these microenvironments represent a major aspect of tumour progression [9–11]. Many carcinomas and predominately breast cancers are characterised by a dense reactive stroma associated with extensive type I collagen (COL1) deposition [12]. A hallmark of the malignant process is the acquisition of an invasive phenotype that allows cancer cells to breach their underlying basement membrane [13,14]. As a consequence, invading carcinoma cells become confronted with the 3D COL1-rich environment of the reactive stroma. To develop metastatic capabilities, these tumour cells must therefore acquire the capacity to negotiate this novel microenvironment [13,15]. It is well established that COL1 acts as a physical barrier for cell migration by enmeshing cells in a dense fibrillar network [16,17]. It also hinders the proliferation of both normal and cancer cells [18–20], thereby acting as an endogenous antigrowth signal. However, tumour progression is not solely governed by the rate of cell proliferation but also by the rate of cell death [13]. Apoptosis is the major source of cell death and an aberrant cell survival resulting from an acquired resistance toward apoptosis represents a prominent hallmark of cancers [13,15]. In this context, COL1 influences the fate of epithelial cell populations by inducing apoptosis [21–23].

The dense fibrillar structure of COL1 is resistant to almost all forms of proteolytic cleavage [24,25]. To date type I collagenolytic activity is limited to a small subset of proteinases belonging to either the cysteine proteinase or matrix metalloproteinase (MMP) families [24,26,27]. MMPs are zinc-dependent endopeptidases that play crucial roles in cancer progression [28–30], not only by degrading physical ECM barriers, but also by regulating the processing of an increasing panel of molecular mediators of signaling events [31,32]. They thus represent the most prominent family of proteinases associated with tumorigenesis [28]. There are 23 different human MMPs described to date, some of which are secreted in the pericellular environment while others are associated with the cell membrane. Membrane-type 1 MMP (MT1-MMP, MMP-14), the best characterized membrane-anchored MMP, is a potent matrix-degrading proteinase that digests a broad spectrum of ECM proteins, including COL1 [33], as well as a number of cell surface-associated proteins [34]. MT1-MMP is overexpressed in many types of tumours including breast carcinomas [35,36], where it constitutes an independent predictor of adverse outcome [37].

We have previously demonstrated that poorly invasive breast adenocarcinoma cells that do not express MT1-MMP undergo apoptosis when embedded in 3D COL1 gels, a model system mimicking the microenvironment encountered by invading carcinoma cells [38]. The expression of MT1-MMP was sufficient to confer cancer cells with the capacity to remodel the matrix and to escape apoptosis. Furthermore, we identified Bcl-2-interacting killer (BIK), a pro-apoptotic member of the Bcl-2 family, as a central mediator of COL1-induced apoptosis [38]. These observations demonstrated that MT1-MMP promoted tumour progression by circumventing collagen-induced apoptosis. To date, however, different questions remain unanswered. Which are the molecular pathways activated by the collagenous microenvironment and how do these pathways lead to the induction of BIK-mediated apoptotic process? How does MT1-MMP alter the cell’s perception of its microenvironment?

Consequently, the aims of the present study were to decipher the molecular mechanisms implicated in (1) the 3D COL1-dependent apoptotic process and (2) the anti-apoptotic function of MT1-MMP.

## Materials and Methods

### Cell lines, culture conditions and reagents

Human breast adenocarcinoma cell lines MCF-7, T47D and ZR-75–1 were obtained from the American Type Culture Collection (Rockville, MD, USA). MCF-7 cells stably transfected with human MT1-MMP cDNA (MT1) or an empty vector (CTRL) were obtained as previously described [38]. To avoid any clone-specific effects, transfected cell lines were generated as polyclonal pools of zeocin-resistant cells (3–5 clones for each pool). All cells were maintained in Dulbecco's modified Eagle's medium (DMEM) supplemented with 10% (v/v) fetal bovine serum (FBS), 100 IU/ml penicillin, 100 μg/ml streptomycin and 2 mM glutamine at 37°C in a humid atmosphere (5% CO2 and 95% air). All culture reagents were purchased from Invitrogen.

### 2D and 3D culture conditions

Type I collagen gels (2 mg/ml) were made from neutralized solutions of native acid-extracted rat tail collagen. Cold Matrigel was diluted twice in serum-free DMEM. For 2D cultures, cell suspensions (12 x 10^4^ cells/well) were plated directly in 12-well plates or on top of a 3D gel of polymerized diluted COL1 (800 μl/well) or a thin layer of COL1 obtained by air drying of polymerized 3D gels overnight at room temperature. Collagen overlays were obtained by casting a COL1 gel over a layer of adherent cells (12 x 10^4^ cells/well). For 3D cultures, cells (2 x 10^5^) were added to the neutralized COL1 or the diluted Matrigel prior to gelling and the gels were cast in 12-well plates (800 μl/well). After polymerization at 37°C, the cell-populated gels were overlaid with 1 ml of DMEM supplemented with 10% FBS.

In some experiments, cell-populated gels were treated with various pharmacological inhibitors at the indicated concentrations: PP2 (5 μM), PP3 (5 μM), saracatinib (1 μM), dasatinib (10 nM), Y-27632 (20 μM), wortmannin (250 nM), LY294003 (10 μM), herbimycin A (500 nM), MK-2206 (2 μM), SB203580 (10 μM), SB239063 (10 μM), blebbistatin (25 μM), BB-94 (1 μM), DDR1-IN-1, a potent and selective DDR1 tyrosine kinase inhibitor [39] was kindly provided by Nathanael Gray, Harvard Medical School, Boston (0.05–10 μM). DMSO (0.1%) was used as a control.

### Quantification of apoptosis

Cells growing on plastic culture plates were detached enzymatically with trypsin-EDTA solution (Invitrogen) and cell embedded in 3D COL1 were recovered after dissolving gels in 2 mg/ml bacterial collagenase (Sigma-Aldrich). Apoptosis was quantified as described previously [38].

### Cell Cycle Analysis

Cells growing on plastic culture plates were detached enzymatically with trypsin-EDTA solution and cells embedded in 3D COL1 were recovered by dissolving gels in 2 mg/ml bacterial collagenase. After gel dissolution, the reaction was stopped with DMEM supplemented with 10% FBS. After washes with cold PBS, collected cells were used for cell cycle analysis by flow cytometry using BD cycle test plus DNA Reagent kit (BD Biosciences, Heidelberg, Germany) according to the manufacturer’s protocol. The samples were filtered through a 35-μm cell strainer and analysed with a FACSCalibur flow cytometer (BD Biosciences). Data were analysed with ModFit LT software.

### RNA purification and microarray hybridization

Total RNA was isolated using TRIzol reagent (Invitrogen) and an RNeasy Mini Kit (Qiagen) as described previously [38]. RNA quantification was performed using a NanoDrop ND-1000 spectrophotometer (NanoDrop Products, Wilmington, DE, USA). The purity and quality of extracted RNA were evaluated using the Experion RNA StdSens Analysis kit (Bio-Rad Laboratories, Hercules, CA). High quality RNA with RNA Quality Indicator (RQI) score greater than 8 was used for microarray experiments. For each experimental condition, RNA samples extracted from 4 independent experiments were pooled.

Gene expression profiling was performed by the GIGA-GenoTranscriptomic plateform (University of Liège) using Illumina’s multi-sample format Human HT-12 v4 BeadChip array that contains more than 47000 probes and profiles twelve samples simultaneously on a single chip (Illumina Inc., San Diego, CA). For each sample, 250 ng total RNA was labelled using a Illumina TotalPrep 96-RNA Amplification kit (Ambion, Austin, TX) according to the manufacturer’s instructions. Briefly, double stranded cDNA was synthesized using T7-oligo (dT) primers and followed by an *in vitro* transcription reaction to amplify cRNA while biotin was incorporated into the synthesized cRNA probe. The cRNA probe was then purified and quantified using a NanoDrop spectrophotometer. Biotinylated cRNA probe was hybridized to the Human HT-12 BeadChip array (Illumina). Labeled cRNA (750 ng) was used for hybridization to each array. The hybridization, washing, and scanning were performed according to the manufacturer’s instructions.

### Microarray data analysis

The arrays were scanned using a BeadArray Reader (Illumina). The microarray images were registered and extracted automatically during the scan according to the manufacturer’s default settings. Data extraction and initial quality control of the bead summary raw data were performed using BeadStudio v3.1.0.0 from Illumina and the Gene Expression module v3.1.7. Variance-stabilizing transformation (vst) [40] and quantile normalisation were performed using the R package lumi in Chipster analysis software [41]. The Illumina probes were re-annotated using Re-annotation and Mapping for Oligonucleotide Array Technologies (ReMOAT) [42] and only probes with good or perfect quality were used. For each experimental condition, tab separated text files with vst transformed and quantile normalised intensities for each probe were exported for further analysis. The mRNA expression dataset has been deposited in the National Center for Biotechnology Information (NCBI) Gene Expression Omnibus (GEO; http://www.ncbi.nlm.nih.gov/geo) and is accessible through GEO Series accession number GSE49595 (http://www.ncbi.nlm.nih.gov/geo/query/acc.cgi?acc=GSE49595). Unsupervised Hierarchical Clustering (HCL), were performed with the software tool of The Institute for Genomic Research (TIGR) MeV (http://www.tigr.org/software/tm4/mev.html) [43]. Euclidian distance and average linkage were used for HCL. Venn analysis was performed using gene symbols.

### Semi-quantitative RT-PCR

For validating microarray data, total RNA isolated for gene expression profiling was used. RT–PCR was performed as described previously [44]. Sequences of forward and reverse primers (Eurogentec, Seraing, Belgium) used are listed in S1 Table.

Methods for RNA interference, western blotting, immunofluorescence microscopy, time-lapse imaging, biological pathway and Gene Ontology term analysis are described in S1 Supplementary Methods, published as supporting information on the *PLoS ONE* web site, www.plosone.org.

### Statistical analysis

All quantitations experiment data are expressed as mean ± standard error of the mean (SEM). Statistical analysis were conducted with GraphPad Prism software (La Jolla, CA, USA) using one or two-way ANOVA where appropriate, followed by Boneferroni’s test or using Kruskal-Wallis followed by Dunn’s test. *p<*0.05 was considered statistically significant.

## Results and Discussion

### Topology and composition of the matrix are important determinants for BIK induction

**Fig 1:**
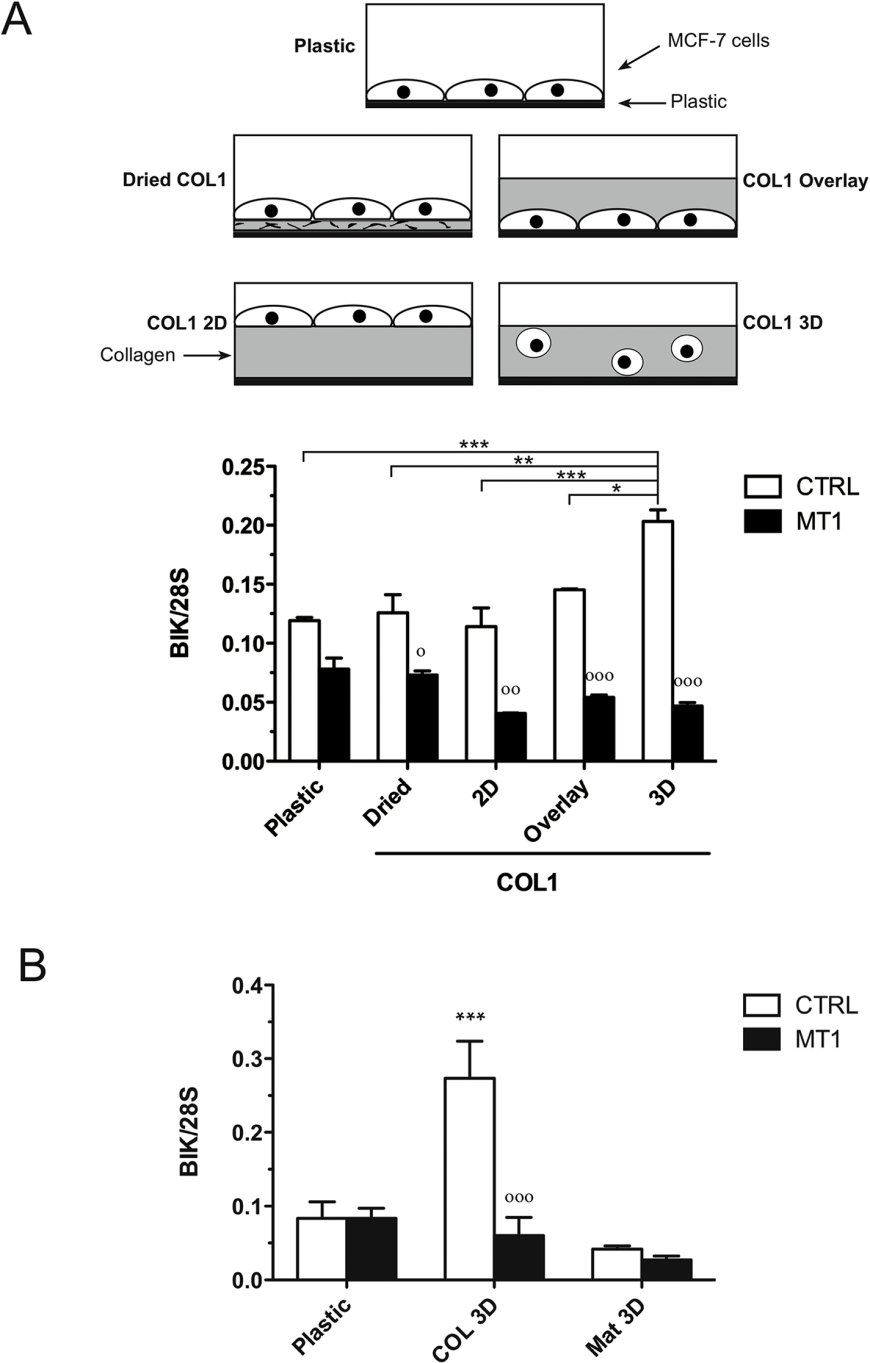
Influence of the topology and composition of the matrix on BIK expression. Control (CTRL) and MT1-MMP (MT1) expressing MCF-7 cells were cultured for 48h in different experimental settings. (**A**) For 2D cultures, the cells were plated directly on plastic (Plastic), on a thin coat of dried COL1 (Dried COL1), on top of a 3D gel of polymerized COL1 (COL1 2D), overlaid with a 3D COL1 gel (COL1 Overlay). For 3D cultures, the cells were embedded into a COL1 gel (COL1 3D). (**B**) CTRL and MT1 MCF-7 cells were cultured on plastic or embedded within 3D COL1 (COL 3D) or 3D Matrigel (Mat 3D). BIK expression was quantified by semi-quantitative RT-PCR. Relative expression levels were obtained after normalization for the 28S rRNA levels. Data are means ± SEM (n = 3). * p<0.05, ** p<0.01, *** p<0.001 MT1 *versus* CTRL; # p<0.05, ### p<0.001 matrix *versus* Plastic (two-way ANOVA with Bonferroni post tests; *, genotype effect; #, matrix effect).

We have previously shown that when poorly invasive luminal-like breast carcinoma cells were switched from conventional 2D cell culture plastic plates to a 3D environment made of native acid-extracted COL1, the expression of BIK, a pro-apoptotic member of the BCL-2 family, was dramatically increased, thereby triggering apoptotic cell death [38]. We first evaluate the influence of matrix dimensionality on COL1-induced apoptosis. Control (CTRL) and MT1-MMP expressing (MT1) MCF-7 human breast cancer cells were cultured for 48 hours in five different experimental settings (Fig. 1) and BIK expression was quantified as a surrogate for apoptosis [38]. The cells were either (a) plated on plastic, (b) plated on a thin coat of dried COL1, (c) plated on top of a 3D COL1 gel, (d) plated on plastic and then overlaid with a 3D COL1 gel or (e) suspended into a 3D COL1 gel. Quantification of BIK mRNA level by semi-quantitative RT-PCR demonstrated that the induction of BIK was restricted to CTRL cells embedded in 3D COL1 (Fig. 1). These observations suggest that the initiation of the apoptotic process required not only the presence of three-dimensionally organized collagen fibrils, but also the interaction of the collagen with the whole cell surface, thereby leading to a loss of cellular polarization. To further evaluate the impact of the microenvironment on BIK induction, control and MT1-MMP expressing MCF-7 cells where embedded in 3D Matrigel, a self-assembling hydrogel mostly composed of laminin and non-fibrillar type IV collagen [45]. Contrary to 3D COL1, 3D Matrigel failed to increase BIK expression in both cell lines (Fig. 1), demonstrating that both the topology and the composition of the matrix are important determinants for BIK induction.

### 3D COL1 induces major alterations in the transcriptome of MCF-7 cells

To obtain a more complete picture of the molecular alterations induced when MCF-7 were switched from 2D Plastic to a 3D COL1 microenvironment, a transcriptomic profiling was carried out. CTRL and MT1 cells were incubated on 2D plastic or within 3D COL1 for 24, 48 and 72 hours. RNA samples from the different experimental conditions were subjected to microarray analysis on the Illumina HT-12 BeadChip array. To minimize the biological variability of the results, RNA samples obtained from 4 independent biological replicates of each experimental condition have been pooled. Box-whisker plots of signal intensities values for the 47,231 probes from each of the 12 arrays before and after normalization showed that the distribution intensities in each data set were similar for all samples (S1 Fig.). Of 47,231 probes represented on the BeadChip, 16,443 (35%) were expressed in at least 1 experimental condition (S2 Fig.). All data have been deposited on the GEO repository under the experimental title: “Genome-wide analysis of control and MMP-14 (MT1-MMP) expressing MCF-7 cells growing in three-dimensional (3D) type I collagen gels versus monolayer cell culture conditions » (accession number: GSE49595). Probes showing a fold change ≥ 1.8 in at least one condition (3D COL1 versus 2D Plastic or MT1 versus CTRL, at any time point) were selected for further analysis. This selection identified 1187 differentially expressed probes (which map to 1061 unique genes) that fulfilled this criterion.

To validate the reproducibility of our microarray data, the mRNA levels of BIK as well as 5 randomly selected genes that were modulated by ≥ 1.8-fold were measured by semi-quantitative RT-PCR. We confirmed the up-regulation of BIK in CTRL cells growing within 3D COL1 and we observed a good correspondence between the modulations detected by semi-quantitative RT-PCR and microarray analysis (S3 Fig.).

**Fig 2:**
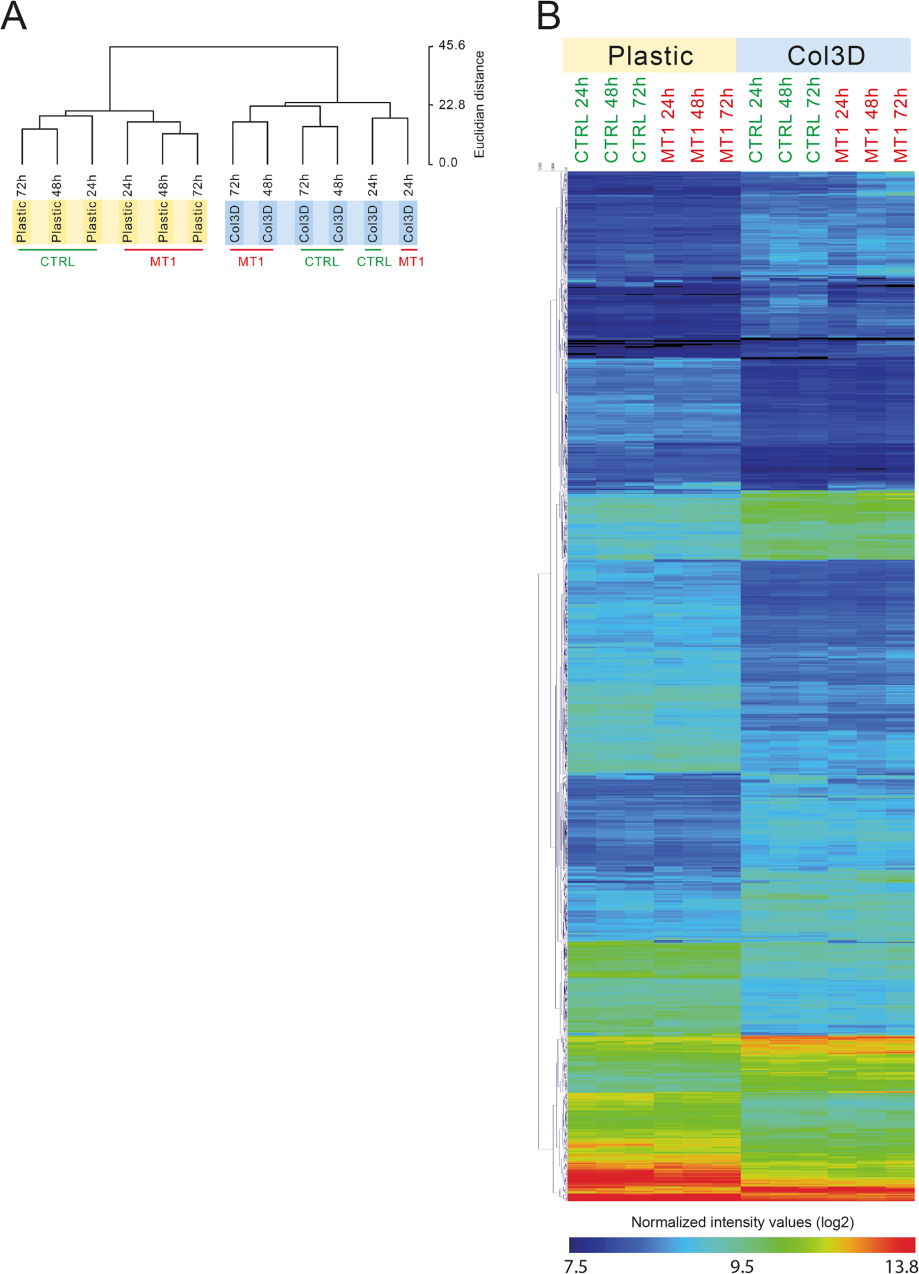
Hierarchical clustering of probes and samples. Control (CTRL) and MT1-MMP (MT1) expressing MCF-7 cells were cultured for 24, 48 and 72h on 2D plastic (Plastic) or within 3D COL1 (Col3D). RNA was extracted from each sample and gene expression values measured using the Illumina Human HT-12 BeadChip array. (**A**) Unsupervised hierarchical clustering of the 12 samples. The length and the subdivision of the branches display the relatedness of the samples. Hierarchical clustering was performed using Euclidian as distance measure and average linkage. A complete version of this hierarchical clustering is available as S2 Fig. (**B**) Heat map representation of normalized signal intensity values (log2) for probes altered by ≥ 1.8-fold (for CTRL versus MT1 or 3D COL1 versus 2D plastic, in at least one time point). Red represents relative expression greater than the median expression level across all samples, and blue represents an expression level lower than the median expression level. The colour intensity represents the magnitude of the deviation from the median. The dendrogram at the left provides a measure of the relatedness of the probe expression profile in each sample.

From this transcriptomic analysis, it was obvious that the cellular microenvironment (2D Plastic versus 3D COL1) has a much stronger impact on gene expression profiles than the expression of MT1-MMP or culture duration. The relationships between the different experimental conditions and probes expression profiles were analysed using unsupervised hierarchical clustering (Fig. 2A and S2 Fig.). The relationship between samples is presented as a dendrogram in which the length of the dendrogram branches is inversely proportional to the relatedness of gene expression patterns. This analysis revealed that the MCF-7 cells (control or MT1-MMP expressing) growing in 3D COL1 clustered together on a single branch of the dendrogram, indicating that they were closely related to each other and were significantly different from the cells plated on 2D plastic, that clustered on a separate branch of the dendrogram (Fig. 2A and S2 Fig.). This analysis also revealed that for each microenvironment (plastic or 3D COL1), the gene expression profiles distinguished cells expressing MT1-MMP (MT1) from control cells (CTRL), except after 24h in 3D COL1, suggesting that the influence of MT1-MMP expression on the transcriptome was less prominent at this specific time point. The gene expression heat map of the 1187 differentially expressed probes showed that the gene expression profile of MCF-7 cells was drastically altered when the cells were shifted from 2D Plastic to 3D COL1 environment (Fig. 2B).

**Fig 3:**
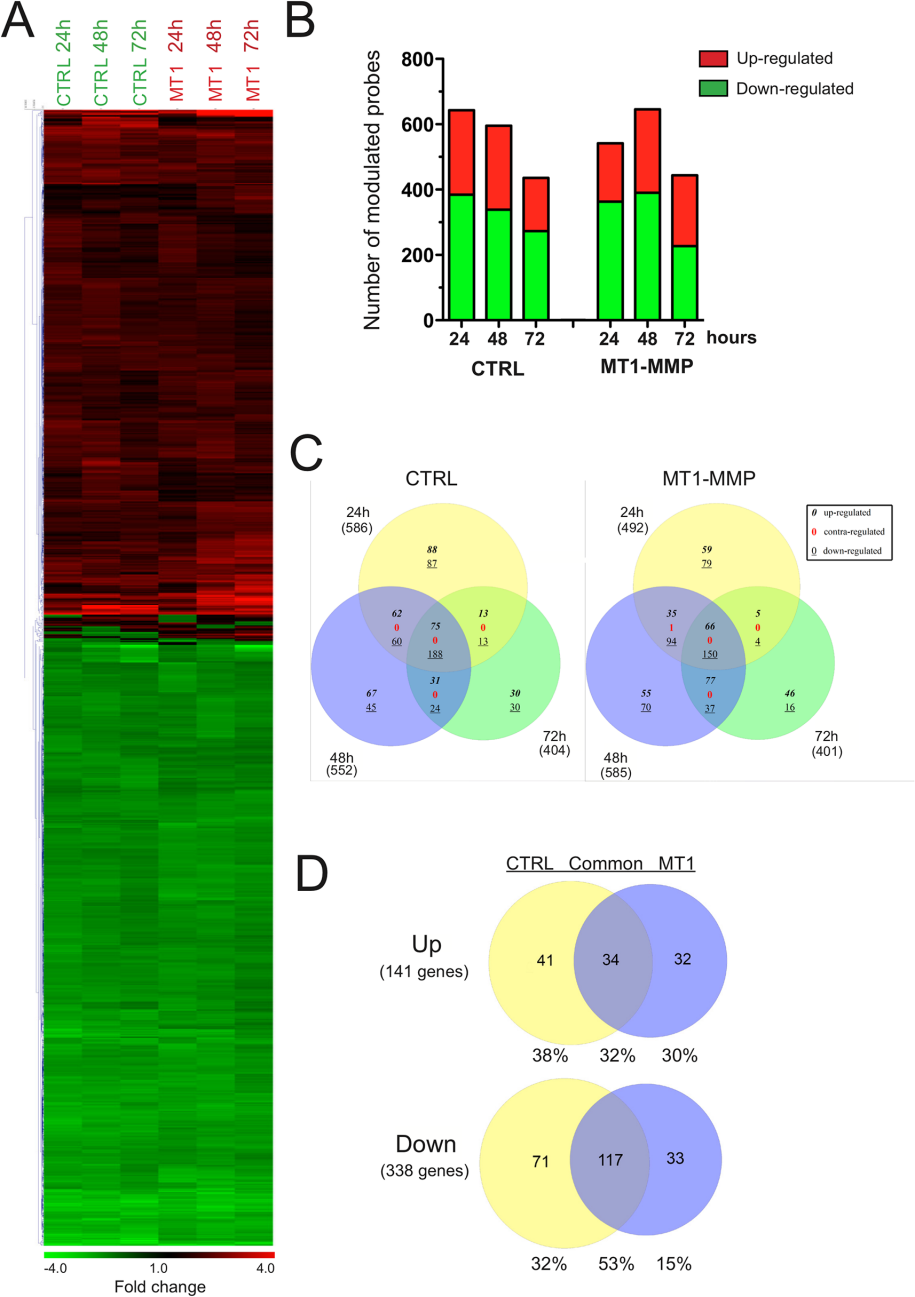
Transcriptomic alterations induced in CTRL and MT1 MCF-7 cells when shifted from 2D plastic to 3D COL1. Control (CTRL) and MT1-MMP (MT1) expressing MCF-7 cells were cultured for 24, 48 and 72h on 2D plastic (Plastic) or within 3D COL1 (Col3D). RNA was extracted from each sample and gene expression values measured using the Illumina Human HT-12 BeadChip array. (**A**) Probes with a fold change ≥ 1.8 in at least one cell type were selected and displayed as a heat map based on unsupervised hierarchical clustering. Each column represents a cell line at a specific time point and each row represents a probe. Red colour indicates genes that were up-regulated and green colour indicates genes that were down-regulated. Black indicates genes whose expression is unchanged in 3D COL1 as compared to 2D Plastic. (**B**) Number of probes modulated in response to the shift from 2D plastic to 3D COL1. (**C**) Venn analysis showing the overlap of genes at the three time points in CTRL and MT1 cells. Numbers in italics, red, and underlined represent up-, contra-, and down-regulated genes, respectively. Numbers in brackets refer to the numbers of genes modulated at each time point. (**D**) Venn diagram analysis of genes similarly modulated at the three time points in CTRL and MT1 MCF-7 cells in response to 3D COL1. Numbers in brackets refer to the numbers of genes modulated at each time point. Percentages represent the proportion of genes present in each area of the diagrams. Venn analyses were performed using gene symbols.

We first focused on the impact of the microenvironment on the transcriptome of MCF-7 cells (Fig. 3A). Culturing CTRL cells in 3D COL1 modulated the expression of 642, 595 and 435 probes after 24, 48 and 72 hours, respectively. A fairly similar number of probes were affected in MT1 cells (541, 645 and 443 probes after 24, 48 and 72 hours, respectively) (Fig. 3B). To determine which biological processes were regulated by 3D COL1, we analysed the Gene Ontology (GO) enrichment in the genes modulated by 3D COL1 in CTRL and MT1 cells. The five most highly-enriched terms for each cell line are shown in Table 1, and the full list is provided in S2 Table. This analysis revealed that 3D COL1 affected very similar biological processes in both CTRL and MT1 cells. For example, the glucose catabolic process was predicted to be increased while the RNA processing as well as the cell cycle were decreased by 3D COL1 at the three time points. This overall similarity in the biological processes affected by 3D COL1 in both cell lines prompted us to determine the global correspondences between the gene modulated after 24, 48 and 72 hours. In that purpose, we used Venn diagrams and investigated the intersections formed (S4 Fig.). At each time point, the majority of the collagen-modulated genes were similarly affected in CTRL and MT1 cells.

**Table 1:** Top 5 significant GO terms enriched in individual lists of 3D COL1-regulated genes.

CTRL			MT1		
GO term	*p*-value	Gene count	GO term	*p*-value	Gene count
**Up-regulated by 3D COL1 after 24h**					
generation of precursor metabolites and energy	3.04E-06	17	generation of precursor metabolites and energy	1.07E-05	12
translational elongation	6.21E-06	10	oxidation reduction	4.57E-04	14
oxidation reduction	2.63E-05	23	glucose catabolic process	6.96E-04	5
alcohol catabolic process	8.36E-05	8	hexose catabolic process	0.00133	5
glucose catabolic process	9.97E-05	7	monosaccharide catabolic process	0.00148	5
**Down-regulated by 3D COL1 after 24h**					
mRNA metabolic process	1.65E-05	20	mRNA metabolic process	4.17E-06	18
RNA processing	1.76E-05	25	regulation of cell cycle	4.27E-06	17
regulation of cell cycle	4.66E-05	18	RNA processing	1.76E-05	21
DNA metabolic process	1.33E-04	22	RNA splicing	6.14E-05	14
cell cycle	2.76E-04	28	DNA metabolic process	6.78E-05	19
**Up-regulated by 3D COL1 after 48h**					
translational elongation	1.19E-04	7	translational elongation	7.50E-04	6
			amine catabolic process	0.0022	5
			alcohol catabolic process	0.0026	5
			glycolysis	0.0044	4
			fructose metabolic process	0.0063	3
**Down-regulated by 3D COL1 after 48h**					
cell cycle	6.90E-06	26	RNA processing	1.48E-05	21
regulation of cell cycle	1.20E-05	16	regulation of cell cycle	1.61E-05	16
RNA processing	1.14E-04	19	cell cycle	3.16E-05	25
mRNA metabolic process	1.65E-04	15	DNA metabolic process	5.81E-05	19
cell cycle process	5.05E-04	18	mRNA metabolic process	5.83E-05	16
**Up-regulated by 3D COL1 after 72h**					
alcohol catabolic process	6.48E-04	5	alcohol catabolic process	3.36E-05	6
hexose metabolic process	0.0024	6	glycolysis	6.15E-05	5
glucose catabolic process	0.0028	4	glucose catabolic process	1.41E-04	5
fructose metabolic process	0.0030	3	hexose metabolic process	2.40E-04	7
generation of precursor metabolites and energy	0.0041	7	hexose catabolic process	2.77E-04	5
**Down-regulated by 3D COL1 after 72h**					
RNA processing	5.25E-05	17	RNA processing	9,52E-06	17
mRNA metabolic process	1.8E-04	13	mRNA metabolic process	5,06E-05	13
RNA splicing	0.00142	10	RNA splicing	5,44E-04	10
response to organic substance	0.00309	16	DNA metabolic process	8,98E-04	13
regulation of cell cycle	0.00399	10	cell cycle	0,00165	16

Genes modulated in the presence of 3D COL1 were analysed by using the functional annotation tool of DAVID for functional enrichment analysis, with the DAVID default population background for Homo sapiens. The significance value associated with each Gene ontology term is a measure of the likelihood that the association between modulated transcripts and a given term is due to random chance. The *p*-value was calculated using the Fisher's Exact test. A complete version of this table is available as S2 Table.

Venn diagram analysis illustrated in Fig. 3C also revealed that 32% (75 up- and 188 down-regulated genes) of the genes whose expression is modulated by 3D COL1 in CTRL cells were common to the three time points and might therefore represent the gene expression signature of the response of CTRL cells to the 3D COL1 microenvironment. Similarly, 27% (66 up- and 150 down-regulated genes) of the genes modulated by 3D COL1 in MT1 cells were common to the three time points (Fig. 3C). The comparison of these two molecular signatures demonstrated that despite their strongly divergent phenotypes in the 3D COL1 microenvironment [38], CTRL and MT1 cells present rather similar gene expression profiles. Indeed, a significant fraction of the gene expression signatures of these two cell types was commonly modulated (32% and 53% were commonly up- and down-regulated, respectively) (Fig. 3D). These similarly regulated genes were accordingly considered as a common signature of transcriptional changes in response to 3D COL1 (S3 Table). To gain insight into the biological processes that were modulated, the 151 genes composing the “3D COL1 core signature” were explored using the core analysis function included in Ingenuity Pathways Analysis (IPA). This analysis revealed that 3D COL1 altered several bio-functions including among others *Cell cycle*, *Cell death/survival*, *Cellular function/maintenance*, *Cellular growth/proliferation* and *RNA post-transcriptional modification* (Table 2).

**Table 2:** Bio-functions associated with the "3D COL1 core signature".

Bio-functions	Functions Annotation	# genes	*p*-value
Cell cycle	cell cycle progression	17	3.64E-03
Cell Death and Survival	cell death	51	2.13E-07
	apoptosis	44	3.31E-07
Cellular Function and Maintenance	cellular homeostasis	21	3.08E-03
	microtubule dynamics	16	6.17E-03
	organization of cytoskeleton	17	1.28E-02
Cellular Growth and Proliferation	proliferation of cells	50	3.69E-06
	proliferation of tumor cell lines	23	7.43E-04
Cellular Movement	invasion of cells	14	1.27E-03
	cell movement	24	1.88E-02
DNA Replication, Recombination, Repair	DNA recombination	4	4.99E-03
	repair of DNA	6	7.44E-03
	formation of spindle fibres	3	8.55E-03
Gene Expression	transcription	28	4.49E-04
Infectious Disease	replication of virus	12	1.00E-03
RNA Post-Transcriptional Modification	processing of RNA	9	1.29E-04
RNA Trafficking	transport of RNA	3	6.88E-03

Bio-function analysis of the dataset of genes similarly regulated by 3D COL1 in both CTRL and MT1 cells. The significance value associated with each category is a measure of the likelihood that the association between modulated transcripts and a given process is due to random chance. The *p*-value was calculated using the right-tailed Fisher's Exact Test. A complete version of this table is available as S7 Table.

Despite the striking different sensitivity of CTRL and MT1 cells towards 3D COL1-induced apoptosis [38], a significant fraction of the core gene signature common to these two cell lines was associated with cell death (51 out of 151 genes) and apoptosis (44 out of 151 genes), suggesting that transcriptional modulations associated with this biological process were not completely abrogated in MT1-MMP expressing cells (Table 2). This assumption is further supported by the observation that the expression of several key players in apoptosis (including BAD, BAG3, BCL2, BCLAF1 and BOK) was similarly modulated by collagen in both CTRL and MT1 cells (S5 Fig.).

### 3D COL1 alters cell cycle progression

As mentioned in Tables 1 and 2, the bioinformatics analysis points to an alteration of several genes involved in cell cycle and proliferation. To measure the impact of 3D COL1 on cell proliferation, we examined cell cycle distribution in MCF-7 cells by measuring DNA content using flow cytometry. CTRL and MT1 cells were cultivated on 2D plastic or within 3D COL1 gels for 24, 48 and 72 hours, nuclei were isolated, fixed, stained using propidium iodide and analysed by FACS. Twenty-four hours after shifting CTRL cells from 2D plastic to 3D COL1 gels, the population of nuclei in G_0_/G_1_ phase was significantly increased at the expense of the G_2_/M phase (S6 Fig. and S4 Table), while the percentage of cells in S phase was not affected. A similar but not significant trend was observed for the MT1 cells. After 48 hours in 3D COL1, the percentages of CTRL nuclei in S and G_2_/M phases were strongly decreased, concomitant with the accumulation of nuclei in G_0_/G_1_ phase (S6 Fig. and S4 Table). A this time point, 3D COL1 did not influence the cell cycle distribution in MT1 cells, except for the G_2_/M phase which was slightly reduced. After 72 hours in 3D COL1, CTRL and MT1 cells exhibited similar cell cycle profiles characterized by a reduction of the percentages of nuclei in both S and G_2_/M phases associated with an increased population in G_0_/G_1_ phase (S6 Fig. and S4 Table). The lack of difference between collagen embedded CTRL and MT1 cells at this specific time point might result from the much higher cellular density reached by the rapidly growing MT1 cells. It is worth noting that these alterations in cell cycle progression induced by 3D COL1 were paralleled by the decreased expression of cyclin-dependent kinases such as CDK1 (also known as CDC2) and CDK6 as well as the increased expression of cyclin-dependent kinase inhibitor 1A (CDKN1A or p21/WAF1) as revealed by our microarray analysis (S7 Fig.). The expression of KIAA0101 (also known as p15/PCNA-Associated Factor 15), a gene shown to promote cell cycle progression [46], was down-regulated by 3D COL1 (S7 Fig.). Collectively, these data indicated that 3D COL1 halted cell cycle progression at G1 or decreased entry in S phase.

### 3D COL1 modifies the expression of genes associated with the organization of the cytoskeleton

Several genes associated with the organization of the cytoskeleton as well as microtubule dynamics were modulated in response to 3D COL1 (Table 2). Among these genes, **γ**-actin (ACTG1), a protein essential for cytoskeletal maintenance [47], angiopoietin-like 4 (ANGPTL4) which is implicated in fibrillar actin reorganization and formation of pseudopodia [48], ADP-ribosylation factor-like 2 (ARL2) which is involved in the polymerization of microtubules [49] and N-myc downstream regulated 1 (NDRG1) shown to inhibit fibrillar actin polymerization and stress fibre formation [50] were up-regulated in both CTRL and MT1 cells in response to 3D COL1 (S8 Fig.). In contrast, the expression of FAT tumour suppressor homolog 1 (FAT1), a member of the cadherin superfamily associated with actin filament disorganization and disturbance of cell-cell contacts [51] and Rho-associated, coiled-coil containing protein kinase 2 (ROCK2) a serine/threonine kinase implicated in the formation of actin stress fibres and focal adhesions [52] were down-regulated in 3D COL1 (S8 Fig.). Important genes implicated as sensors of the microenvironment including integrin **β**_1_ (ITGB1), **β**_2_ (ITGB2), **α**_2_ (ITGA2), **α**_5_ (ITGA5) and **α**_v_ (ITGAV), discoidin domain receptor tyrosine kinase 1 (DDR1) and E-cadherin (CDH1), albeit not fulfilling all the criteria to be included in the 3D COL1 core signature, displayed significant alterations in response to 3D COL1. ITGB1, ITGAV and CDH1 were down-regulated (S9 Fig.) while ITGB2, ITGA5 and DDR1 were up-regulated in 3D COL1 (S9 Fig.). A similar down-regulation of ITGB1 expression has been previously observed in HT1080 cells upon inclusion in 3D COL1 gels and was associated with a decreased cellular volume [53]. These observations support the concept that shifting the cells from 2D Plastic to 3D COL1 induced a complex reprogramming of genes implicated in cellular architecture as well as in interactions with the microenvironment.

### 3D COL1 alters the expression of genes associated with RNA processing

The analysis of the 3D COL1 core signature with DAVID revealed a significant enrichment of Gene Ontology terms associated with *RNA processing*, *RNA splicing*, *ribonucleoprotein complex*, *nuclear lumen* and *spliceosome* (Table 3). A detailed analysis of our microarray data revealed that the expression of 69% of the genes encoding heterogeneous nuclear ribonucleoparticle (hnRNP) proteins represented in the HT-12 BeadChip array (18 probes out of the 24 present in our microarray, corresponding to 9 genes out of 13) was decreased by a factor of at least 1.8-fold in 3D COL1 (S10 Fig.). HnRNPs have been shown to associate with pre-mRNAs, forming large hnRNP-RNA complexes [54]. HnRNP proteins are multifunctional, participating in all crucial aspects of RNA processing, including pre-mRNA splicing, mRNA export, localization, translation and stability [55]. They also regulate the expression of several RNA binding protein transcripts and cancer-associated transcripts [56]. Decreased HNRNPA2B1 expression induces apoptosis of cancer cells by increasing the formation of the pro-apoptotic Bcl-x(s) [57]. HNRNPK and HNRNPH1 also exert anti-apoptotic functions [58,59]. Beyond their implications in the regulation of apoptosis, hnRNPs such as HNRNPA2B1, HNRNPD and HNRNPM are also involved in RNA post-transcriptional modifications including the control of mRNA decay [60] and alternative splicing [61]. The spliceosome, a complex of RNA and many protein subunits, is a major actor in RNA post-transcriptional modifications through its pre-mRNA splicing activity [62]. Several transcripts encoding components of the spliceosome (DDX5, HNRNPA2B1, HNRNPH1, HNRNPR, PDCD7, PNN, SRSF1, SRSF2, SRSF5) were down-regulated in 3D COL1. The recognition of splicing sites is dependent on the protein composition of the spliceosome [63]. Therefore, the deregulated expression of the genes coding for spliceosome components could modify the composition of the spliceosome architecture, with an impact on the splicing process. A change in the splicing process may in turn affect the cell at all biochemical levels, from the transcriptome to the proteome. DICER1, another key RNA processing enzyme implicated in the maturation of short precursor microRNAs into mature microRNAs [64], was also down-regulated in 3D COL1 (data not shown), supporting a potential alteration of miRNA biogenesis.

**Table 3:** Gene ontology terms associated with the 3D COL1 signature.

Gene Ontology Id	Gene Ontology Name	# genes	*p*-value
**GO:0030529**	ribonucleoprotein complex	15	2.53E-05
**GO:0005654**	nucleoplasm	19	7.44E-05
**GO:0031981**	nuclear lumen	23	7.88E-04
**GO:0031974**	membrane-enclosed lumen	27	7.97E-04
**GO:0043233**	organelle lumen	26	0.00137
**GO:0070013**	intracellular organelle lumen	25	0.00225
**GO:0005681**	spliceosome	6	0.00301
**GO:0030530**	heterogeneous nuclear ribonucleoprotein complex	3	0.00691
**GO:0016607**	nuclear speck	5	0.00722
**GO:0016604**	nuclear body	6	0.00827
**GO:0043228**	non-membrane-bounded organelle	29	0.02111
**GO:0005813**	centrosome	6	0.02567
**GO:0042470**	melanosome	4	0.02853
**GO:0005815**	microtubule organizing center	6	0.0402

Genes included in the 3D COL1 core signature were analysed by using the functional annotation tool of DAVID for functional enrichment analysis, with the DAVID default population background for *Homo sapiens*. The significance value associated with each Gene ontology term is a measure of the likelihood that the association between modulated transcripts and a given term is due to random chance. The *p*-value was calculated using the Fisher's Exact test. A complete version of this table is available as S8 Table.

### MT1-MMP expression minimally interferes with 3D COL1-induced transcriptomic alterations

In contrast to the dramatic effect of 3D COL1 on the transcriptome of MCF-7 cells, the expression of MT1-MMP had only a modest influence on the gene expression profile of cells growing either on 2D plastic or within 3D COL1 (S11 Fig.).

**Fig 4:**
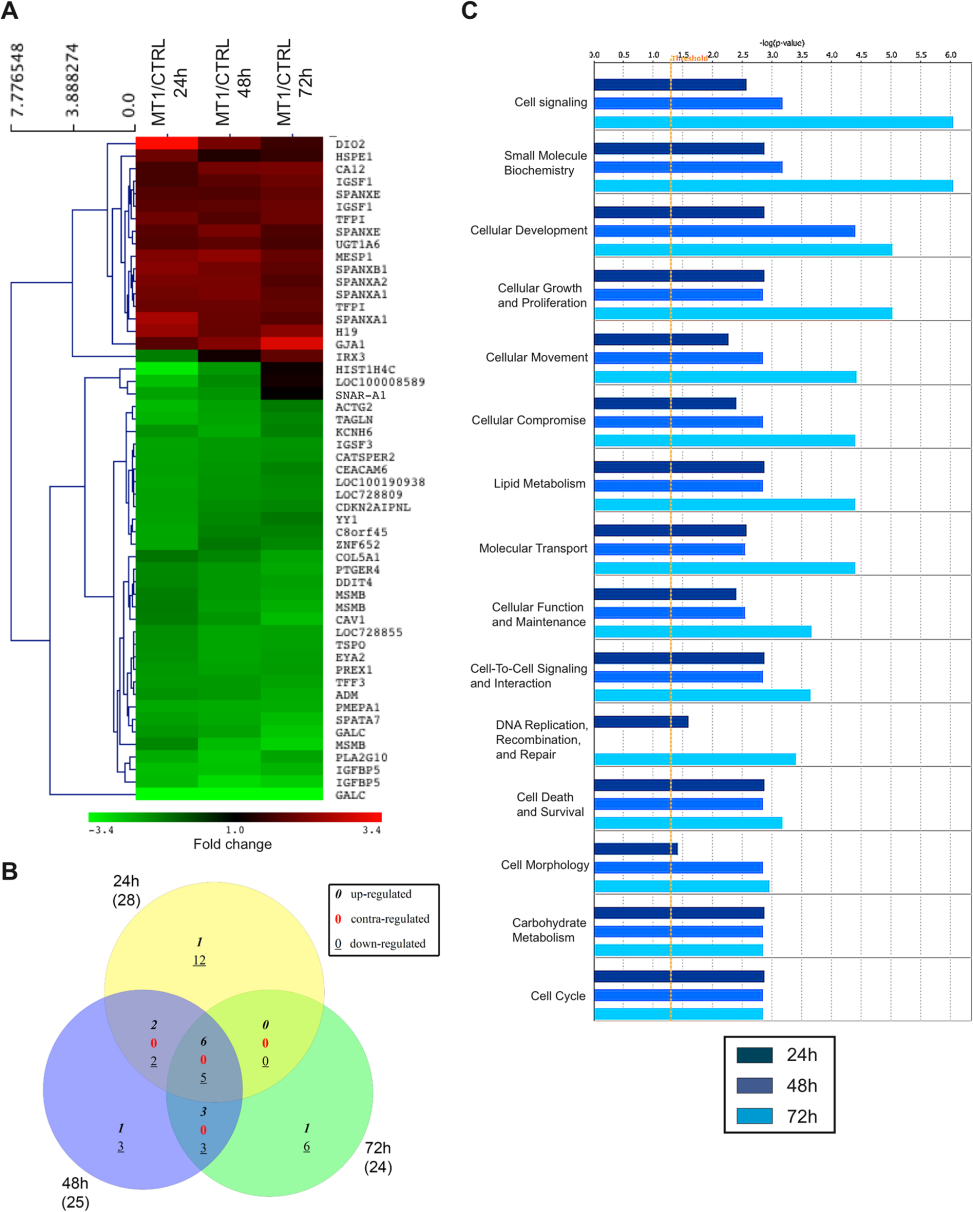
Transcriptomic alterations induced by MT1-MMP in MCF-7 cells maintained on 2D plastic. Control (CTRL) and MT1-MMP (MT1) expressing MCF-7 cells were cultured for 24, 48 and 72h on 2D plastic. RNA was extracted from each sample and gene expression values measured using the Illumina Human HT-12 BeadChip array. (**A**) Probes with a fold change ≥ 1.8 between CTRL and MT1 cells in at least 1 point in the time course were selected and displayed as a heat map based on unsupervised hierarchical clustering. Red colour indicates genes that were up-regulated and green colour indicates genes that were down-regulated. Black indicates genes whose expression is unchanged in MT1 cells as compared to CTRL cells. (**B**) Venn diagram showing the overlap of modulated genes at the three time points. Numbers in italics, red, and underlined represent up-, contra-, and down-regulated genes, respectively. Numbers in brackets refer to the numbers of genes modulated at each time point. Venn analyses were performed using gene symbols. (**C**) Bio-function analysis of the dataset of genes differentially regulated upon MT1-MMP expression was performed in IPA. The significance is expressed as a negative log of p-value, which was calculated using the right-tailed Fisher's Exact Test. The orange line represents the threshold p value of 0.05.

On 2D plastic, 17 probes (corresponding to 14 genes) were up-regulated, while 36 probes (corresponding to 32 genes) were down-regulated for at least 1 point in the time course upon expression of MT1-MMP (Fig. 4A). Venn diagram analysis of modulated genes revealed that only 11 genes (6 up- and 5 down-regulated) were consistently regulated at 24, 48 and 72 hours (Fig. 4B and S5 Table). IPA analysis revealed that the MT1-modulated genes were implicated in bio-functions including among others *Cell signalling*, *Small molecule biochemistry* and *Cellular development* (Fig. 4C).

**Fig 5:**
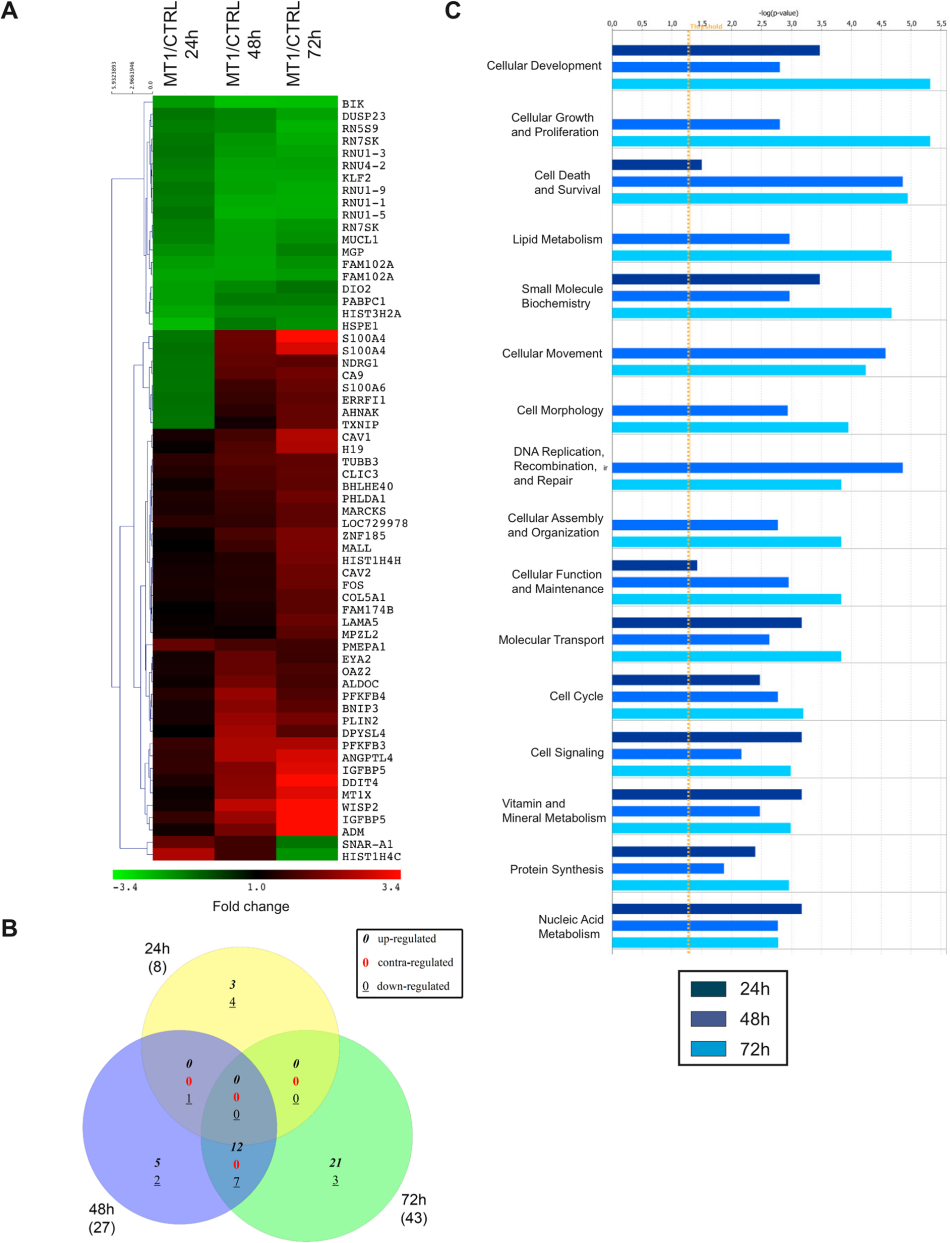
Transcriptomic alterations induced by MT1-MMP in MCF-7 cells embedded in 3D COL1. Control (CTRL) and MT1-MMP (MT1) expressing MCF-7 cells were cultured for 24, 48 and 72h in 3D COL1. RNA was extracted from each sample and gene expression values measured using the Illumina Human HT-12 BeadChip array. (**A**) Probes with a fold change ≥ 1.8 between CTRL and MT1 cells in at least 1 point in the time course were selected and displayed as a heat map based on unsupervised hierarchical clustering. Red colour indicates genes that were up-regulated and green colour indicates genes that were down-regulated. Black indicates genes whose expression is unchanged in MT1 cells as compared to CTRL cells. (**B**) Venn diagram showing the overlap of modulated genes at the three time points. Numbers in italics, red, and underlined represent up-, contra-, and down-regulated genes, respectively. Numbers in brackets refer to the numbers of genes modulated at each time point. Venn analyses were performed using gene symbols. (**C**) Bio-function analysis of the dataset of genes differentially regulated upon MT1-MMP expression was performed in IPA. The significance is expressed as a negative log of p-value, which was calculated using the right-tailed Fisher's Exact Test. The orange line represents the threshold p value of 0.05.

**Fig 6:**
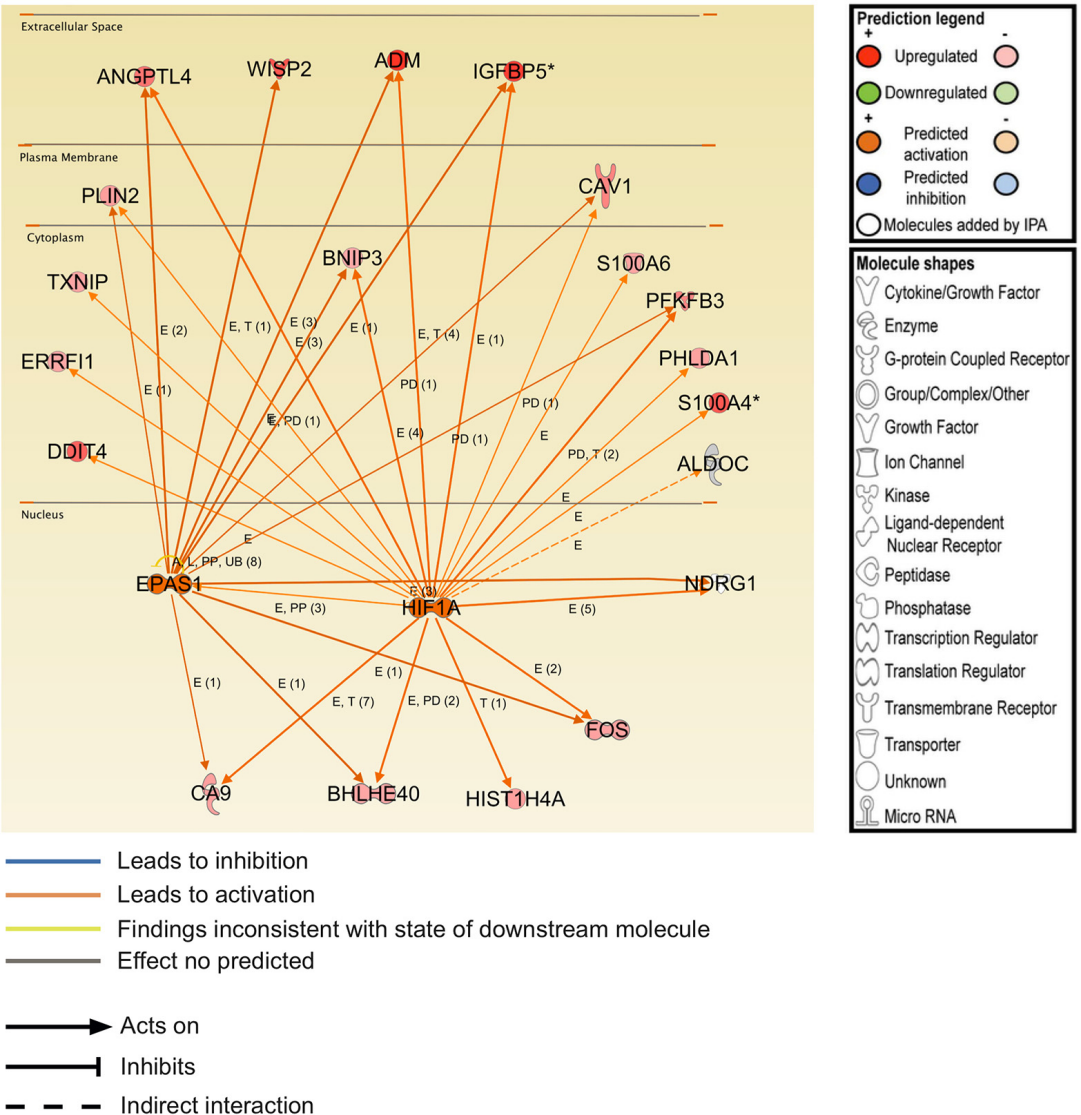
Transcriptional regulators of the MT1-MMP modulated genes in 3D COL1. The IPA Upstream regulator analysis identified transcription factors with direct actions on differentially expressed target genes. The different molecules are presented by cellular localization. HIF1A and EPAS1 were predicted to be activated (or to have increased activity) in MT1 cells relative to CTRL cells. Genes in red and green are up- and down-regulated in response to MT1-MMP expression, respectively. E: expression; PD: protein-DNA binding; RB: regulation of binding; T: transcription.

Within 3D COL1, MT1-MMP up-regulated the expression of 42 probes (corresponding to 41 genes) while it down-regulated 20 probes (corresponding to 17 genes) (Fig. 5A). After 24 hours in 3D COL1, only 8 genes were transiently affected by MT1-MMP (Fig. 5A and B and S6 Table), suggesting that the impact of MT1-MMP expression on the transcriptome of MCF-7 cells growing within 3D COL1 was extremely limited at this early time point. This observation corroborates the unsupervised hierarchical clustering of samples that failed to discriminate the transcriptome of CTRL and MT1 cells growing in 3D COL1 after 24 hours (Fig. 2A). Consequently, no core signature common to the three time points investigated could be identified. At 48 and 72 hours, the number of genes affected by MT1-MMP increased as a function of time. Global functional analysis with IPA revealed that the genes modulated by MT1-MMP in 3D COL1 were implicated in bio-functions including *Cellular development*, *Cellular growth & Proliferation* and *Cell death & Survival* (Fig. 5C). IPA predicted a decrease in *Cell death of tumor cell lines* (z-score-2.12; *p* = 0.0009; 9 molecules: ADM, DRG1, BIK, S100A4, KLF2, DDIT4, MT1X, BNIP3 and IGFBP5), thus confirming a correlation between the transcriptomic alterations induced by MT1-MMP and the reduced apoptosis observed in these cells [38]. The upstream regulator analysis by IPA identified HIF1A and EPAS1 (also known as HIF2A) as major regulators of the genes up-regulated in MT1 cells after 48 and 72 hours in 3D COL1 (Fig. 6). These transcription factors, which belong to the Hypoxia Inducible Factor (HIF) family of basic helix-loop-helix transcription factors, were able to control the expression of 44% and 46% of the genes modulated by MT1-MMP after 48 and 72 hours, respectively. Nevertheless neither HIF1A nor EPAS1 were transcriptionally regulated in our samples (data not shown), suggesting that MT1-MMP promotes the transcriptional activity of these factors at a post-transcriptional level. Accumulating evidence has proved that HIF1A activity is triggered under normoxia by different factors including hormones, growth factors, low pH, reactive oxygen species and mechanical stress [65,66]. The intracytoplasmic domain of MT1-MMP has been shown previously to stimulate HIF-1 [67] and EPAS1/HIF2 [68] activities independently of oxygen levels. In contrast to the data reported by Sakamoto and co-workers [67], we observed that the up-regulation of HIF1A target genes (such as ANGPTL4, S100A4 and CA9) in 3D COL1-embedded MT1 cells was reverted upon treatment with a synthetic MMP inhibitor (BB-94), demonstrating that the catalytic activity of MT1-MMP was required for these modulations (data not shown). The ability of MT1-MMP to up-regulate HIF1A/HIF2A target genes is specific to the 3D COL1 microenvironment since the culture in 3D Matrigel failed to recapitulate the up-regulation of ANGPTL4, S100A4 and CA9 expression observed in 3D COL1 (data not shown).

Among HIF-target genes that were up-regulated in 3D COL1 embedded MT1 cells, ANGPTL4 (S12 Fig.) has been shown to confer cancer cells with anoïkis resistance [69] and to prime breast cancer cells for lung metastasis [70]. CA9 (S12 Fig.) was implicated in several specific biological processes critical for cancer progression, including pH regulation and cell survival, adhesion, migration and invasion, and the acquisition of chemo and radioresistant properties [71]. NDRG1 (S12 Fig.) has been shown to promote epithelial-to-mesenchymal transition [72], cell adhesion, proliferation [73], and to inhibit apoptosis [74]. S100A4 (S12 Fig.) was involved in migration and invasion of cancer cells [75]. Finally, BNIP3 (S12 Fig.) was associated with protective autophagy, cell survival [76,77] and enhanced tumour growth [78].

In contrast to the up-regulated genes, no upstream regulator able to control the genes down-regulated in 3D COL1-embedded MT1 cells was identified. Among the down-regulated genes, BIK was the only gene directly implicated in apoptosis [38,79].

**Fig 7:**
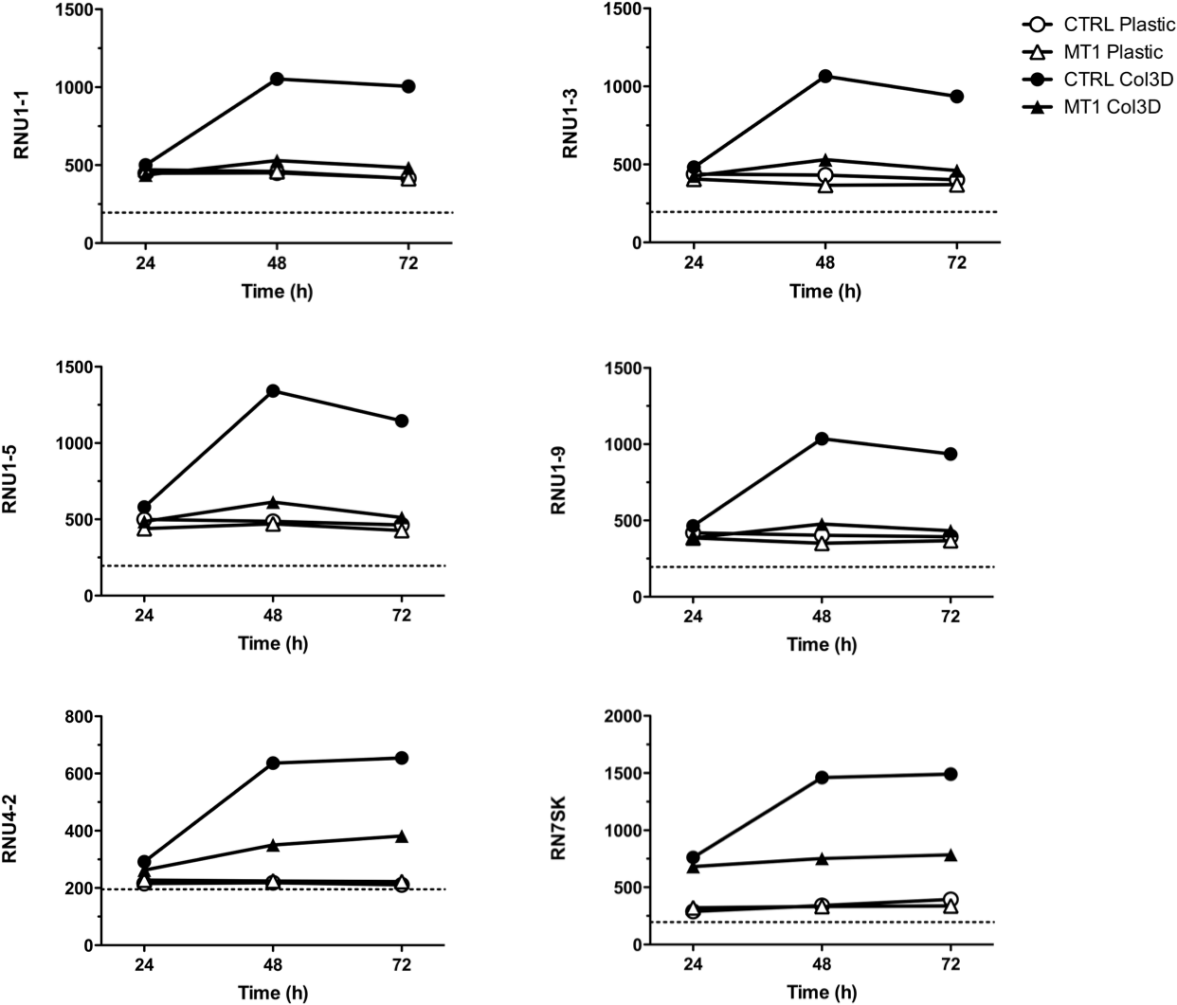
3D COL1 modulates the expression of small nuclear RNAs (snRNAs)-coding genes. Control (CTRL) and MT1-MMP (MT1) expressing MCF-7 cells were cultured for 24, 48 and 72h on 2D plastic (Plastic) or within 3D COL1 (Col3D). RNA was extracted from each sample and gene expression values measured using the Illumina Human HT-12 BeadChip array. Microarray data were expressed as fluorescence intensities. Dashed line represents the background fluorescence.

Genes encoding small nuclear RNAs (snRNAs) were over-represented in the down-regulated genes after 48 and 72 hours (S6 Table and Fig. 7). The controlled expression of snRNAs is critically important to cellular homeostasis [80]. These genes displayed an expression profile very similar to BIK (compare Fig. 7 and S3 Fig.): both were up-regulated in CTRL cells by 3D COL1 as from 48h and were only marginally affected in MT1 cells. Among these snRNAs, RNU1–1 (also known as U1 snRNA) and three of its variants (RNU1–3, RNU1–5 and RNU1–9) were affected. RNU1–1 represents an essential component of the spliceosome [62], but it also plays regulatory roles in gene expression by inhibiting polyadenylation as well as by protecting nascent transcripts from premature cleavage/polyadenylation events occurring at cryptic poly(A) sites [81]. RN7SK (also known as 7SK RNA), another snRNA down-regulated in MT1 cells, is a key actor in the regulation of gene expression in response to stress signals [82] and it inhibits RNA polymerase II-mediated transcriptional elongation, thereby contributing to the regulation between gene expression and silencing [83]. The potential influence of the down-regulation of these snRNAs in MT1 cells exposed to 3D COL1 is currently unclear; however, it can be hypothesized that their up-regulation in apoptotic CTRL cells (Fig. 7) contributes to the apoptotic process. Collectively, these data support the concept that MT1-MMP might foster the survival of MCF-7 cells in 3D COL1 by inhibiting pro-apoptotic genes such as BIK while up-regulating several HIF1A/HIF2A target genes that have been shown to promote the escape from an hostile environment.

### MT1-MMP modulates cellular morphology in 3D COL1 microenvironment

To delineate the timeframe in which the interactions between cells and 3D COL1 are critical for the induction of BIK expression and hence for apoptosis initiation, MCF-7 cells were plated on 2D plastic or suspended in 3D COL1 for increasing period of time (ranging between 0.5–48 hours). RT-PCR analysis revealed that BIK mRNA levels increased, albeit not statistically significantly, after 8 hours in the presence of collagen. A statistically significant up-regulation of BIK expression was observed from 24 hours onwards (S13 Fig.). A similar kinetic of induction was observed in ZR-75–1 cells (S13 Fig.), another breast carcinoma cell line sensitive to 3D COL1-induced apoptosis [38]. This late onset contrasts with the modulation of other transcripts such as ITGB1, which was detected as early as 30 min after collagen polymerization (S13 Fig.). These observations suggest that critical interactions between cells and 3D COL1 took place early during the first 24 hours of culture.

**Fig 8:**
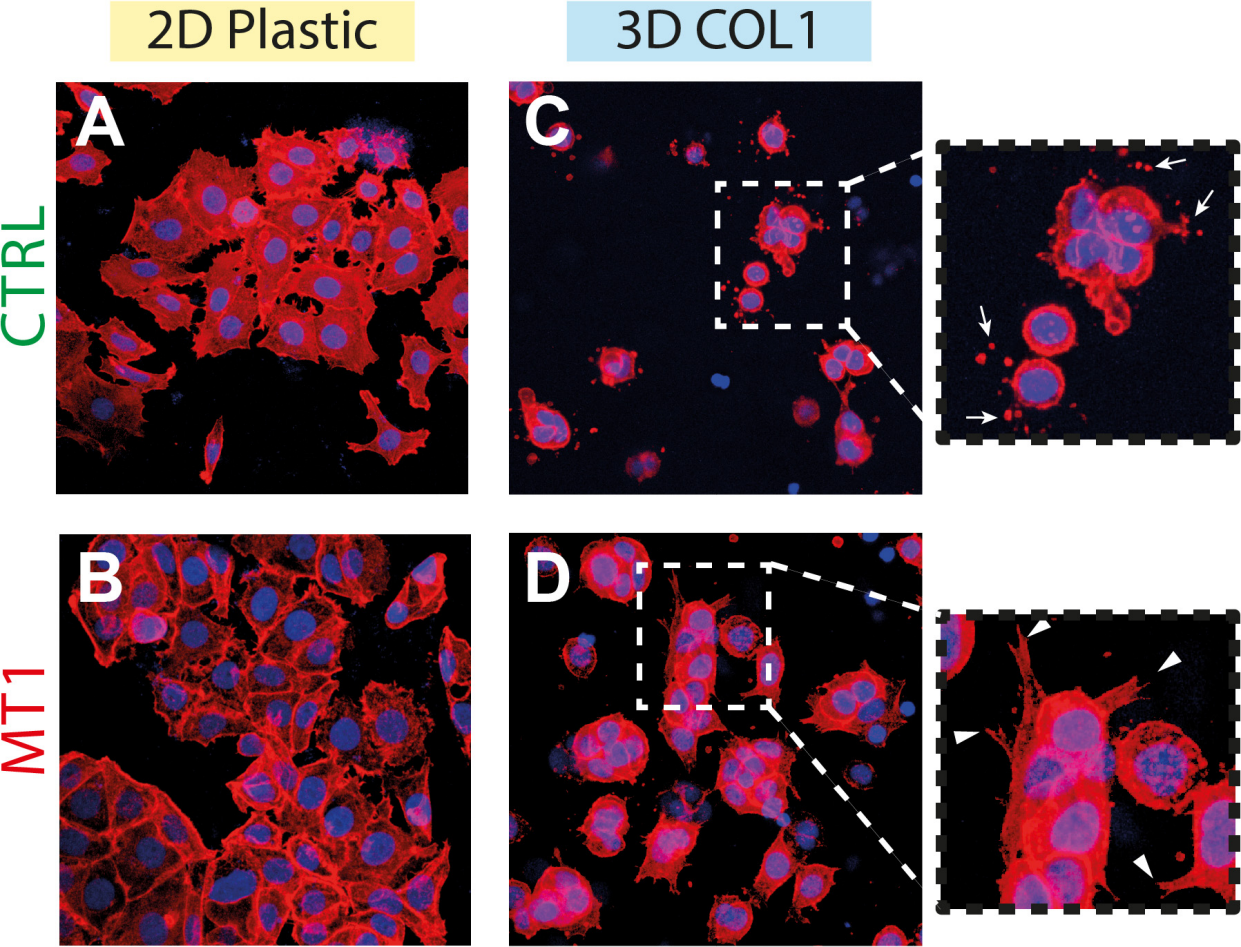
Growth conditions determine cell morphology. Representative maximum intensity projections of confocal z-stacks immunofluorescence images of control (CTRL) and MT1-MMP expressing (MT1) MCF-7 cells cultivated in 2D culture plates (A, B) or embedded in 3D COL1 (C, D). F-actin (red) and DRAQ5 (blue). *Insets*: higher magnification of 3D COL1 embedded cells. *Arrows*, actin-rich vesicles surrounding CTRL cells; *arrowheads*, cytoplasmic protrusions extending from MT1 cells into the COL1 gel.

We next characterized the physical interactions between the cells and the collagen gel during this restricted timeframe. In agreement with the transcriptomic data revealing the altered expression of genes involved in the organization of the cytoskeleton (Table 2) as well as genes coding for receptors of ECM components (S9 Fig.), we observed a significant modification of cell morphology upon shifting MCF-7 cells from 2D plastic to 3D COL1 (Fig. 8). Cells growing on 2D plastic displayed a flattened morphology with classical lamellipodia/pseudopodia (Fig. 8A). In contrast, cells embedded within 3D COL1 exhibited a compacted morphology characterized by a thin cytoplasmic ring surrounding the nucleus (Fig. 8B). While the majority of MT1 cells readily extend cytoplasmic protrusions throughout the collagen matrix, CTRL cells remained trapped in a compact, spherical configuration with most cells surrounded by small actin-rich vesicles (Fig. 8B, insets). These actin-rich vesicles were left behind as a consequence of the retraction of filipodium-like protrusions tipped with small lamellipodia, which were actively exploring the microenvironment (S14 Fig. and S1 Movie). These protrusions were positive for paxillin (S15 Fig.), a major component of focal adhesion complexes formed in 3D collagen gels [84], suggesting that these structures anchored the cells to the collagen fibrils. Paxillin was also detected in the small vesicles left behind after the retraction of the protrusions (S15 Fig., inset). This observations support the concept that CTRL cells inefficiently released their adhesion from the collagen fibrils when protrusions retracted, thereby leading to the accumulation of collagen-associated vesicles around the cells. In the presence of MT1-MMP, most cells extended larger pseudopodia-like protrusions and displayed a higher motility than CTRL cells. The retraction of the protrusions formed by MT1 cells was seldom associated with the appearance of collagen-associated vesicles (S2 Movie). MT1 cells cultured in 3D COL1 in the presence of a synthetic MMP inhibitor (BB-94) phenocopied the morphology and retraction responses exhibited by CTRL cells (S3 Movie), demonstrating the requirement for the catalytic activity of MT1-MMP in the establishment of the larger pseudopodia-like protrusions. In contrast, the treatment with BB-94 did not alter the behaviour of CTRL cells (S4 Movie). These observations suggest that in the absence of MT1-MMP-dependent proteolytic activity, MCF-7 cells were entrapped within a 3D mesh of collagen fibrils. Accordingly, they maintained a spherical configuration and were continuously extending and retracting thin protrusions through the collagen fibrils to probe their microenvironment. Through its capacity to sever COL1 fibrils [19], MT1-MMP allowed the formation of larger protrusions and increased the overall cellular motility. The nearly complete lack of residual vesicles surrounding MT1 cells might similarly result from the MT1-MMP-dependant proteolytic remodelling of collagen fibrils and/or from the cleavage of cell surface collagen receptors thereby enabling the release of the collagen-anchored protrusions. It is well established that MT1-MMP is able to shed an array of cell surface proteins including known collagen receptors such as CD44 [85] or DDR1 [86].

### 3D COL1-mediated BIK induction is mediated by Src family kinases

**Fig 9:**
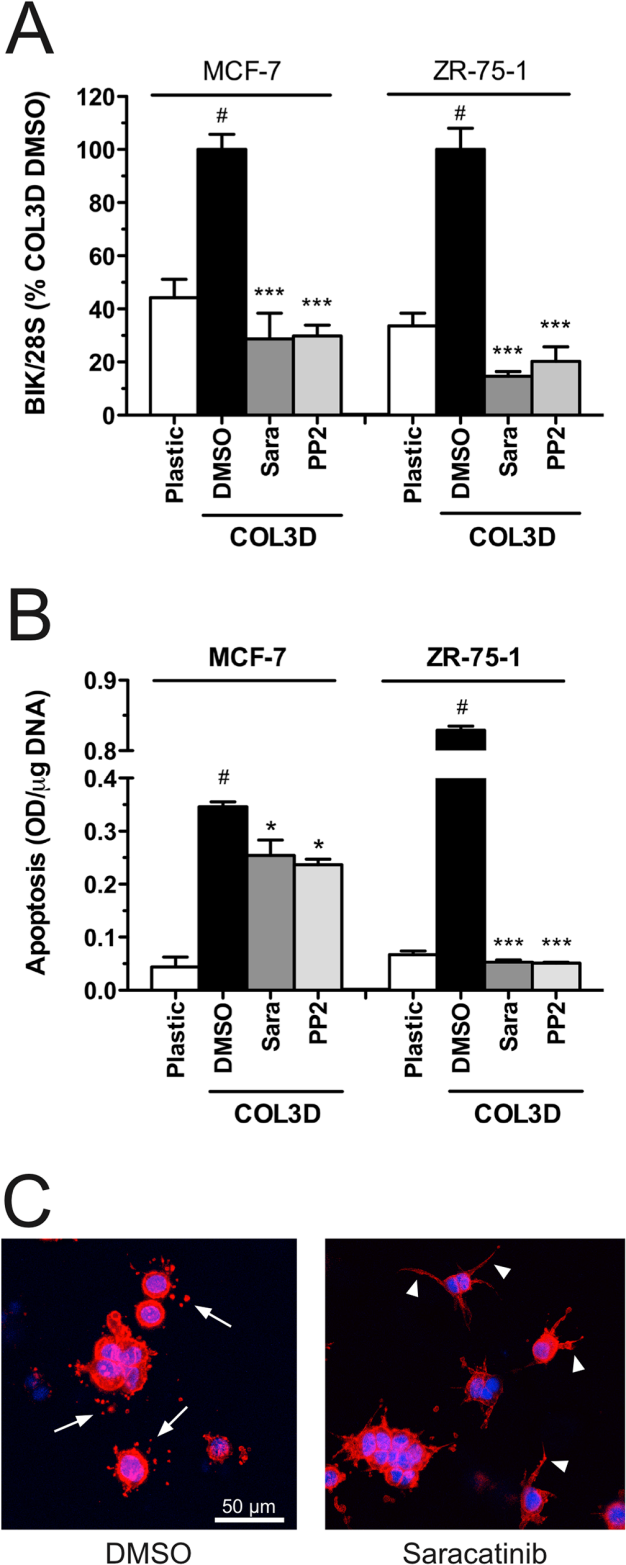
Influence of Src Family Kinase inhibitors on 3D COL1-embedded MCF-7 and ZR-75–1 cells. MCF-7 and ZR-75–1 cells were cultured for 48h on 2D plastic (Plastic) or within 3D COL1 (COL3D) in the presence of PP2 (5 μM), saracatinib (1 μM) or vehicle (DMSO 0.1%). (**A**) RNA was extracted from each sample and BIK mRNA levels were quantified by semi-quantitative RT-PCR. Relative expression levels were obtained after normalization for the 28S rRNA levels. (**B**) Apoptosis was quantified by Cell Death Detection ELISA^PLUS^. Data are means ± SEM (n = 3). ^#^ p<0.01 Col3D *versus* Plastic; * p<0.05, *** p<0.001 treatment *versus* vehicle (one-way ANOVA analysis with Boneferroni post test). (**C**) Representative maximum intensity projection of serial confocal optical sections through MCF-7 cells embedded in 3D COL1 gels and treated with saracatinib (1 μM) or vehicle for 48h. F-actin (red) and DRAQ5 (blue). *Arrows*: actin-rich vesicles; *Arrowheads*: un-retracted cytoplasmic protrusions.

To identify the signalling pathways implicated in 3D COL1-mediated up-regulation of BIK, we tested the effect of pharmacological inhibitors of different pathways upon COL1-induced BIK expression. MCF-7 cells were exposed to the inhibitors immediately after collagen polymerization and BIK expression was quantified after a 48h incubation period (Table 4). 3D COL1-induced BIK mRNA expression was partially inhibited upon treatment with SB203580, an inhibitor of p38 MAPK. However, a more specific p38 MAPK inhibitor (SB239093; [87]) failed to confirm this inhibition, suggesting that p38 MAPK might not be implicated in this signalling pathway. Blebbistatin, a specific inhibitor of non-muscle myosin II, reduced by 50% the expression of BIK in 3D COL1-treated cells, supporting a role for actin-myosin contraction in this process. A robust inhibition of BIK induction was observed upon treatment with herbimycin A, a Src family kinase (SFK) inhibitor [88] (Table 4). SFKs represent a family of non-receptor tyrosine kinases that includes 11 members [89]. The diverse functions of SFKs involve the regulation of cell growth, survival, proliferation, differentiation, adhesion to matrix and a wide range of molecular signalling networks [89]. Our microarray analysis revealed that three SFKs (c-Src, Yes and Frk) were detected in MCF-7 cells (data not shown). To further characterize the contribution of SFKs in the induction of BIK by 3D COL1, MCF-7 and ZR-75–1 cells were treated with more specific SFK inhibitors. Treatment with PP2, saracatinib (Fig. 9A) and dasatinib (10 nM, data not shown) completely prevented the 3D COL1-induced BIK mRNA expression in the two cell lines. The negative control compound PP3 has a similar structure with PP2 but does not affect SFK kinase activity. Treatment with PP3 did not alter BIK expression in either MCF-7 or ZR-75–1 cells (107±16% and 110±12% relative to DMSO-treated cells). We have previously demonstrated that a reduced BIK expression is associated with a lower level of apoptosis [44]. In agreement with this observation, a limited, although statistically significant, decrease in apoptosis was detected in PP2 and saracatinib-treated MCF-7 cells in 3D COL1 when compared with vehicle-treated cells (Fig. 9B). These inhibitors reduced more extensively apoptosis in ZR-75–1 cells (Fig. 9B) than in MCF-7 cells, despite inducing a comparable decrease in BIK mRNA levels. The reason for this difference is unclear but could result from cell-dependent differences in the sensitivity towards pro-apoptotic inducers such as BIK. In addition to preventing apoptosis, SFK inhibitors also altered the morphology of 3D COL1-embedded cells. In contrast to vehicle-treated MCF-7 cells (S5 Movie), PP2 (S6 Movie) and saracatinib (Fig. 9C) treatments both promoted the extension of filipodium-like protrusions and prevented their rupture thereby avoiding the pericellular accumulation of actin-rich vesicles. Collectively, these observations demonstrate that SFK inhibitors prevented the 3D COL1-mediated induction of BIK and stabilized the interactions between cellular protrusions and the collagen fibres.

**Table 4:** Pharmacological inhibition of 3D COL1-induced BIK expression in MCF-7 cells.

Inhibitors	Targets	Concentrations (μM)	BIK expression (% DMSO)
**MK-2206**	Akt	2	68±22 (n = 2)
**SB203580**	p38 MAPK	10	52±11 (n = 2)
**SB239063**	p38 MAPK	10	124±1 (n = 2)
**SP600125**	JNK	10	142±8 (n = 2)
**U0126**	MEK1/2	10	121±12 (n = 2)
**Wortmannin**	PI3K	0.25	160±38 (n = 2)
**Ly294002**	PI3K	10	112±8 (n = 2)
**Blebbistatin**	Non-muscle myosin II	25	49±10 (n = 3)
**Y-27632**	ROCK	20	74±34 (n = 5)
**Herbimycin A**	SFK	0.5	28±19 (n = 3)

Despite their higher specificity towards SFKs, PP2, dasatinib and saracatinib inhibit other tyrosine kinases not belonging to SFKs. Among these kinases, the DDR1 and DDR2 have been recently identified as off-target kinases of these inhibitors [90–92]. DDRs play key roles in cancer progression by regulating the interactions of tumour cells with the collagenous matrices. Among the various proteins that act as collagen receptors, DDRs are unique because of their specific activation in response to collagen [93]. They act as collagen sensors, transducing signals that regulate key cellular functions including tissue morphogenesis and cell differentiation [93]. As opposed to most receptor tyrosine kinase, the kinetic of DDR activation is slow and sustained, requiring several hours to reach maximum phosphorylation [94], suggesting that this receptor may be involved in mediating long-term signals. This slow kinetic is consistent with the late onset of BIK induction observed in 3D COL1 (S13 Fig.).

### 3D COL1-induced apoptosis is dependent on DDR1 tyrosine kinase activity

**Fig 10:**
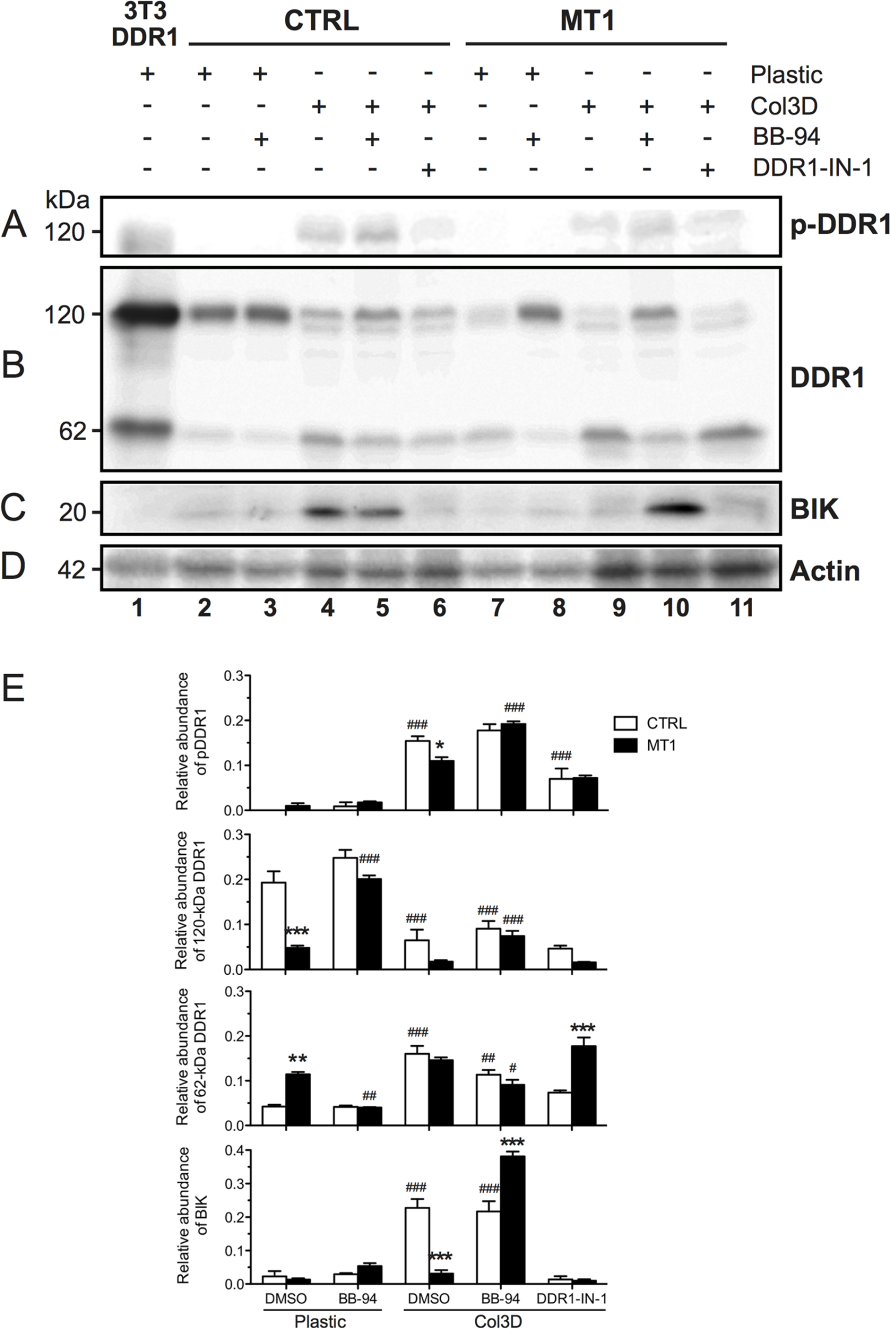
3D COL1 and MT1-MMP regulate DDR1 activation and cleavage. Control (CTRL) and MT1-MMP (MT1) expressing MCF-7 cells were cultured for 24h on 2D plastic (Plastic) or within 3D COL1 (Col3D) in the presence (+) of BB-94 (1 μM), DDR1-IN-1 (1 μM) or vehicle (DMSO 0.1%). 3D COL1 gels were mechanically disrupted and lysed in RIPA buffer. Lysates were resolved by reducing 8% SDS-PAGE followed by immunoblot analysis. Blots were probed with phospho-DDR1 (Tyr792) antibody (*A*) and then reprobed with antibodies directed against the cytosolic juxtamembrane domain of DDR1 (*B*), BIK (*C*) or **β**-actin (*D*), as a loading control. Relative abundances of phosphorylated DDR1, 120-kDa full-length, 62-kDa C-terminal forms of DDR1 and BIK were quantified and normalized with respect to **β**-actin expression. Data are means ± SEM (n = 3). ^#^ p<0.05, ^##^ p<0.01, ^###^ p<0.001 treatment *versus* vehicle-treated cells plated on plastic; * p<0.05, *** p<0.001 MT1 *versus* CTRL cells (one-way ANOVA analysis with Boneferroni post test) (*E*). Lysates of 3T3 cells transfected with human DDR1b cDNA [101], which contains both 120-kDa full-length and 62-kDa C-terminal DDR1 species were included as a positive control.

Our transcriptomic analysis revealed that DDR1 was expressed by the MCF-7 cells (Fig. 6B), while DDR2 was not (data not shown). To examine the influences of 3D COL1 and MT1-MMP on DDR1 activation, CTRL and MT1 MCF-7 cells were incubated on 2D plastic or within 3D COL1 for 24 hours and analysed for DDR1 phosphorylation using a phospho-specific DDR1 antibody directed against the activation loop phosphotyrosine of the DDR1 kinase domain. As shown in Fig. 10A, the phosphorylation of the 120-kDa full-length DDR1 was only detectable in cells growing in 3D COL1 (Fig. 10A, *lanes 4–6* and *9–11*). The abundance of activated DDR1 was reduced, to 71±5% (mean ± SEM, n = 3) of CTRL cells when MT1-MMP was expressed (Fig. 10A, *lanes 4* and *9* and Fig. 10E). BB-94 treatment of MT1 cells restored DDR1 phosphorylation levels similar to those observed in vehicle-treated CTRL cells, while it had no significant impact on DDR1 activation in these latter cells (Fig. 10A, *lanes 5* and *10 and* Fig. 10E), suggesting that MT1-MMP impairs DDR1 activation through its catalytic activity.

Others have shown that MT1-MMP negatively regulates DDR1 activity by shedding its extracellular domain [86]. When CTRL MCF-7 cells, were cultured on plastic (Fig. 10B, *lane 2*), DDR1 was detected as a major 120-kDa form, which corresponds to the full-length receptor, and a minor 62-kDa fragment, which represents a kinase-proficient membrane-anchored C-terminal fragment lacking the extracellular domains implicated in collagen interactions [86]. MT1-MMP expression reduced the level of 120-kDa species to 25±1% of CTRL cells. The reduced level of full-length DDR1 was paralleled by an increase of the 62-kDa form (Fig. 10B, *lanes 2* and *7* and Fig. 10E). This MT1-MMP-mediated DDR1 cleavage was strongly attenuated upon treatment with BB-94 (Fig. 10B, *lanes 7* and *8* and Fig. 10E), thus demonstrating the requirement for the catalytic activity of this proteinase.

Shifting the CTRL cells from plastic to 3D COL1 clearly altered the relative abundance of the DDR1 species, resulting in a 3-fold decrease of the 120-kDa and 4-fold increase of the 62-kDa species (Fig. 10B, *lanes 2* and *4* and Fig. 10E). In this 3D environment, an additional ∼110-kDa DDR1 form was detected (Fig. 10B, *lanes 4–6* and *9–11*). The nature of this DDR1 species is uncertain, but it may represent a differentially glycosylated form of the receptor [86]. When 3D COL1-embedded MCF-7 cells expressed MT1-MMP, a further 3-fold decreased in the level of full-length DDR1 was observed. In contrast, the level of the 62-kDa form remained unchanged (Fig. 10B, *lanes 4* and *9* and Fig. 10E). While BB-94 nearly completely reverted the MT1-MMP-mediated decrease of full-length DDR1 level, it decreased only slightly the abundance of the 62-kDa species in both CTRL and MT1 cells (Fig. 10B, *lanes 5* and *10* and Fig. 10E). These data suggest that embedding MCF-7 cells, which are lacking endogenous MT1-MMP, within 3D COL1 promoted the processing of full-length DDR1 through a BB-94-insensitive process. This ligand-induced receptor cleavage might represent a negative feedback mechanism to protect the cell against receptor over-stimulation.

**Fig 11:**
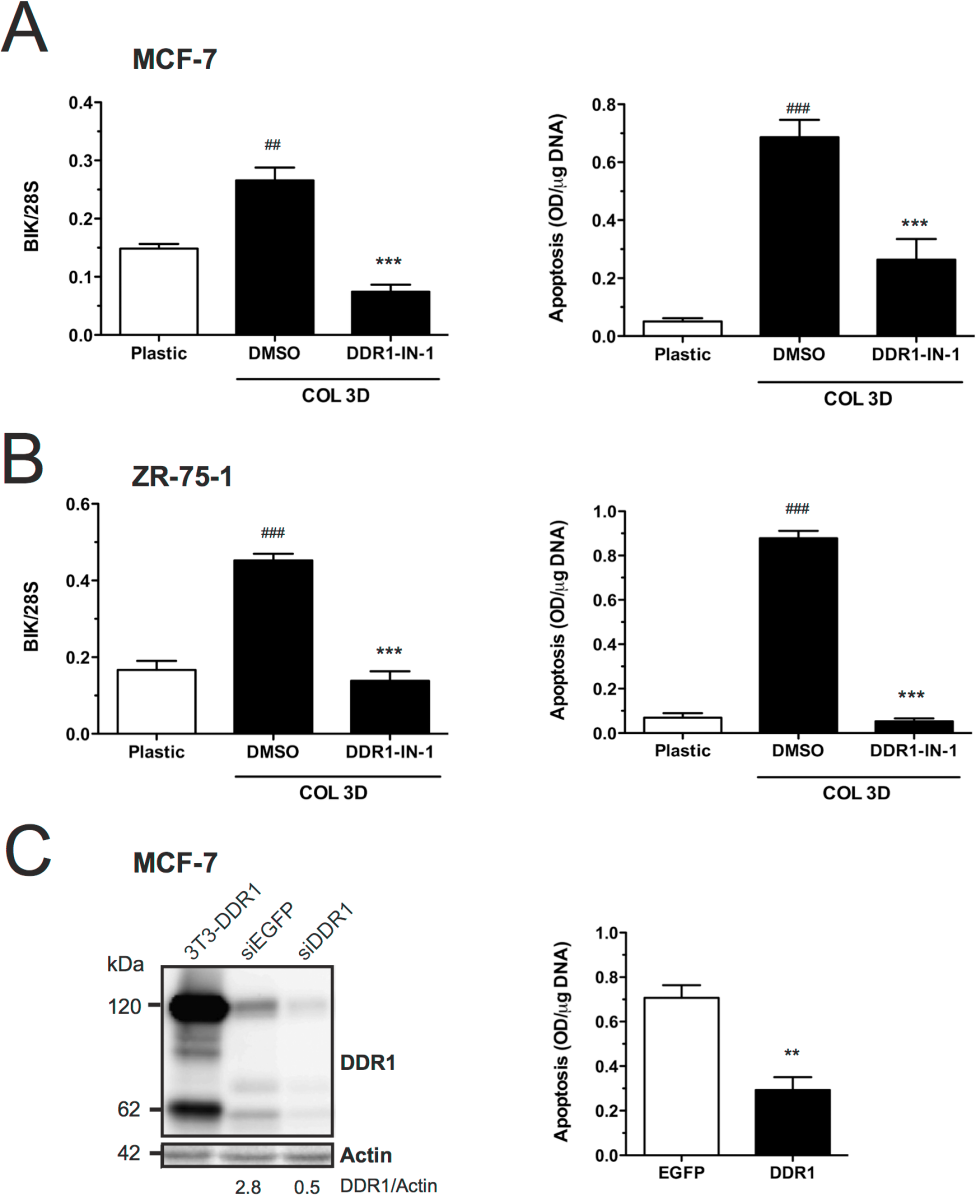
Inhibition of DDR1 tyrosine kinase activity prevents the 3D COL1-mediated induction of BIK. MCF-7 (**A**) and ZR-75–1 (**B**) cells were cultured for 48h on 2D plastic (Plastic) or within 3D COL1 (COL3D) in the presence of DDR1-IN-1 (1 μM) or vehicle (DMSO 0.1%). RNA was extracted from each sample and BIK mRNA levels were quantified by semi-quantitative RT-PCR. Relative expression levels were obtained after normalization for the 28S rRNA levels. (**C**) MCF-7 cells were pre-treated with EGFP or DDR1 esiRNAs for 48h and cultured within 3D COL1 during 24h. esiRNA efficacy was analysed by western blotting. Blots were probed with an antibodies directed against the cytosolic juxtamembrane domain of DDR1 or **β**-actin (*D*), as a loading control. Relative abundances of DDR1 were quantified and normalized with respect to **β**-actin expression. Lysates of 3T3 cells transfected with human DDR1b cDNA [101], which contains both 120-kDa full-length and 62-kDa C-terminal DDR1 species were included as a positive control. Apoptosis was quantified by Cell Death Detection ELISA^PLUS^. Data are means ± SEM (n = 3). ^##^ p<0.01, ^###^ p<0.001 Col3D *versus* Plastic; ** p<0.01, *** p<0.001 treatment *versus* vehicle (one-way ANOVA analysis with Boneferroni post test).

To investigate the potential implication of DDR1 kinase activity in collagen-mediated BIK induction, 3D COL1-embedded MCF-7 cells were treated with DDR1-IN-1, a potent and selective DDR1 tyrosine kinase inhibitor devoid of inhibitory activity against SFKs [39]. DDR1-IN-1 (1μM) efficiently decreased DDR1 phosphorylation in 3D COL1-embedded CTRL cells (Fig. 10A, *lanes 4* and *6* and Fig. 10E). A similar decrease of DDR1 activation was observed in MT1 cells, however, due to their lower initial level of phosphorylated DDR1, the inhibitory effect of DDR1-IN-1 failed to reach statistical significance (Fig. 10A, *lanes 9* and *11* and Fig. 10E). Furthermore, DDR1-IN-1 inhibited the collagen-mediated BIK induction at both mRNA and protein levels, thereby protecting CTRL cells from apoptosis (Fig. 11A and Fig. 10C, *lanes 4* and *6*, respectively). A similar inhibitory effect of DDR1-IN-1 on DDR1 phosphorylation, BIK induction and apoptosis was also observed in 3D COL1-embedded ZR-75–1 cells (Fig. 11B and S16 Fig.).

To further confirm the implication of DDR1 during collagen-induced apoptosis, MCF-7 cells were pre-treated during 48h with DDR1 or EGFP endoribonuclease-prepared siRNAs (esiRNAs) before being incubated within 3D COL1 gels for 24h. As shown in Fig. 11C, DDR1 esiRNAs efficiently down-regulated the expression of DDR1 at the protein level, concomitantly inhibiting apoptosis by about 60% (Fig. 11C).

In contrast to SFK inhibitors, DDR1-IN-1 had no influence on the fate of the cytoplasmic protrusions (data not shown), suggesting that the impact of SFK inhibitors on 3D-COL1-embedded cells was not entirely mediated through the unspecific inhibition of DDR1 activity. This assumption is supported by the reported occurrence of DDRs-SFKs interactions during DDR-initiated signalling [93].

## Conclusions

**Fig 12:**
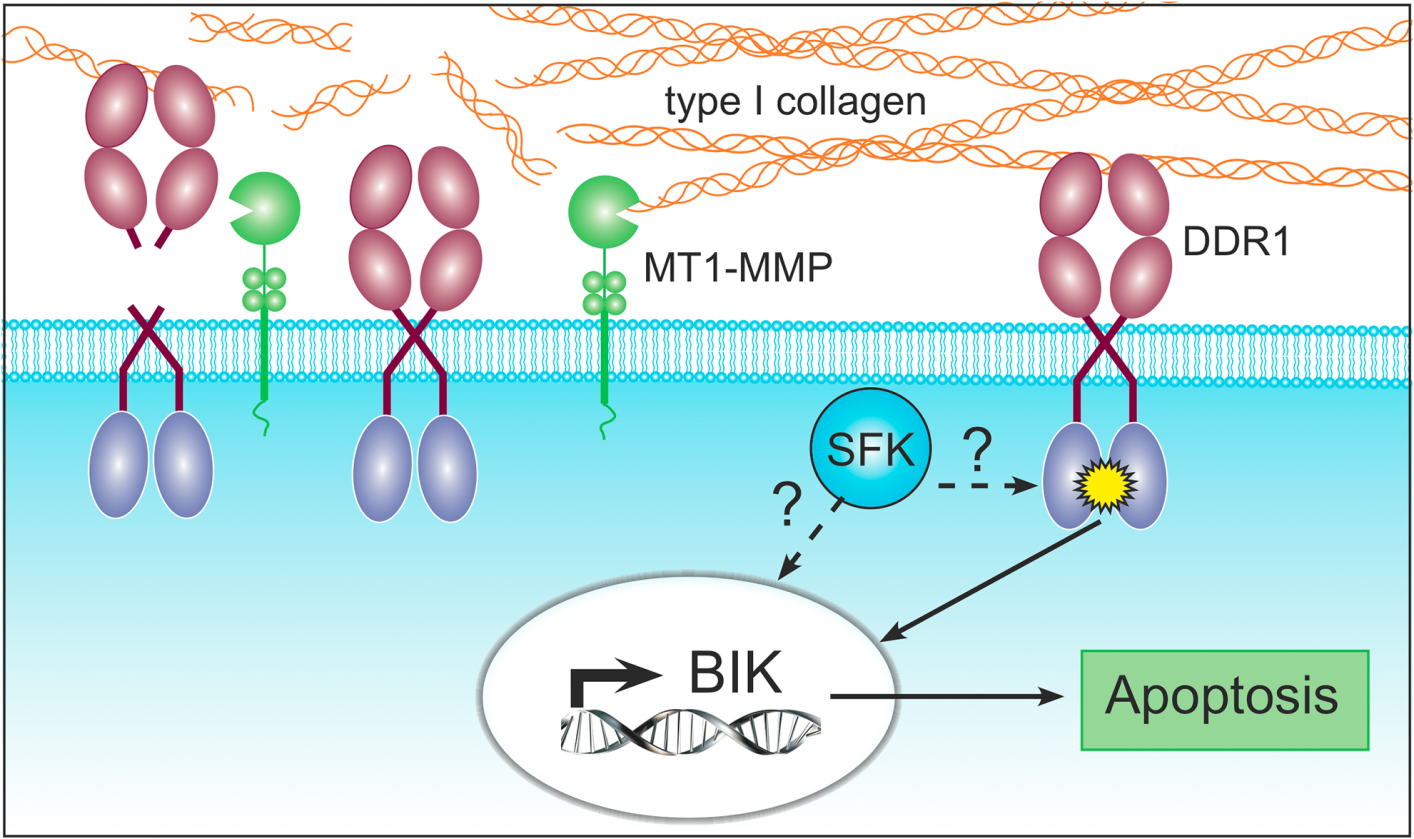
Schematic drawing of 3D COL1-initiated apoptotic process. In the absence of MT1-MMP, triple helical type I collagen activates DDR1 (*yellow star*). Activated DDR1-triggers a signalling pathway culminating in the transcriptional up-regulation of BIK and apoptosis. The exact implication of Src family kinase (SFK) members remains unclear (*question marks*). Catalytically active MT1-MMP can cleave type I collagen, preventing its recognition by DDR1. MT1-MMP is also able to shed the extracellular domain of DDR1. Both cleavages impair DDR1 activation, thereby preventing BIK up-regulation and apoptosis.

Our data demonstrate that the growth of poorly invasive luminal-like breast cancer cells (represented here by the MCF-7 and ZR-75–1 cell lines) in a 3D COL1 microenvironment has profound impacts on several aspects of cell behaviour including cell morphology and gene expression, resulting in the induction of an apoptotic program. We identify DDR1, a collagen receptor tyrosine kinase, as a key sensor that monitors the cellular microenvironment and triggers apoptosis through the induction of BIK (Fig. 12). In contrast with current knowledge [95–97], we demonstrate for the first time a pro-apoptotic function for this collagen-activated receptor. Our results also reveal that the catalytic activity of MT1-MMP impairs this DDR1-initiated apoptotic program. However, the mechanisms by which MT1-MMP interferes with DDR1-initiated signalling remain unclear. These might involve among others: (1) the cleavage of native collagen fibres, which are no longer recognized by DDR1 [93]; (2) the shedding of the extracellular domain of DDR1, which reduces access of the ligand to its cognate receptor and (3) the alteration of the repertoire of DDR1 interacting partners implicated in intracellular signalling.

We have previously reported that in breast cancer cell lines, the induction of BIK by 3D COL1 is observed in the non-invasive cell lines, which retain epithelial features, while the invasive mesenchymal-like cell lines, which underwent an epithelial-to-mesenchymal transition, are resistant [38]. Interestingly, the epithelial-to-mesenchymal transition program that endows epithelial cells with enhanced migratory, invasive and metastatic potential [98] is associated with a down-regulation of DDR1 [99] and an increased expression of MT1-MMP [100], suggesting that the opposite regulation of these two molecules is critical to allow invasive cancer cells to cope with the collagen-rich tumour microenvironment. These observations also emphasize the relevance of using MT1-MMP targeting agents to hamper breast cancer cell dissemination through the collagenous tumour stroma.

## Supporting Information

S1 Fig Quality assessment of microarray data. Box plot depicting the difference among microarray expression datasets from each sample before (A) and after (B) normalization by variance-stabilizing transformation and quantile normalisation performed using the R package lumi in Chipster analysis software. Boxes show the 25^th^ and 75^th^ percentiles in the distribution of log-transformed (log base 2) intensities. The median is the horizontal bar in the middle of the box.
(TIF)

S2 Fig Heat map representation of normalized signal intensity values (log2) for the 16,443 probes expressed in at least 1 experimental condition. Red represents relative expression greater than the median expression level across all samples, and blue represents an expression level lower than the median expression level. The colour intensity represents the magnitude of the deviation from the median. The dendrogram at the left provides a measure of the relatedness of the probe expression profile in each sample. The dendrogram at the top provides a measure of the relatedness of the 12 samples.
(PDF)

S3 Fig Validation of differentially expressed genes by semi-quantitative RT-PCR. Six differentially expressed genes revealed by microarray analysis (A-F) were validated by semi-quantitative RT-PCR (G-L). Microarray data were expressed as fluorescence intensities. Dashed line represents the background fluorescence. For semi-quantitative RT-PCR, relative expression levels were obtained after normalization for the 28S rRNA levels. Data are means ± SEM (n = 4).
(TIF)

S4 Fig Venn diagram showing the overlap of genes with a fold change ≥ 1.8 in response to 3D COL1 at the three time points in CTRL and MT1 cells. Numbers in italics, red, and underlined represent up-, contra-, and down-regulated genes, respectively. Numbers in brackets refer to the numbers of genes modulated at each time point. Percentages represent the proportion of genes present in each area of the diagrams.
(TIF)

S5 Fig Expression of apoptosis-related genes similarly modulated by 3D COL1 in CTRL and MT1 cells. Microarray data were expressed as fluorescence intensities. Dashed line represents the background fluorescence.
(TIF)

S6 Fig Cell cycle analysis of MCF-7 cells cultivated for up to 72 hours on 2D plastic or within 3D COL1. For fluorescence-activated cell sorting (FACS) analysis, control (CTRL) and MT1-MMP expressing (MT1) MCF-7 cells were cultured during 24, 48 and 72h on Plastic or within 3D COL1. Nuclei were isolated and stained with propidium iodide buffer followed by cell sorting analysis. (**A**) The acquired FACS data were analysed by ModFit LT software. (**B**) The results of FACS analysis are presented as mean (±SEM) for four independent experiments. The detailed statistical analysis for each group is illustrated in S4 Table. (**C**) The percentage of cells in S phase is shown. Data are means ± SEM (n = 4). * p<0.05, *** p<0.001 MT1 *versus* CTRL; # p<0.05, ### p<0.001 Col3D *versus* Plastic (two-way ANOVA with Bonferroni post tests); *, genotype effect; #, matrix effect).
(TIF)

S7 Fig Expression of cell cycle-associated genes similarly modulated by 3D COL1 in CTRL and MT1 cells. Microarray data were expressed as fluorescence intensities. Dashed line represents the background fluorescence.
(TIF)

S8 Fig Expression of cytoskeleton-associated genes modulated by 3D COL1 in CTRL and MT1 cells. Microarray data were expressed as fluorescence intensities. Dashed line represents the background fluorescence.
(TIF)

S9 Fig Modulation of genes implicated in cell-cell and cell-ECM interactions by 3D COL1 in CTRL and MT1 cells. The genes were (**A**) down-regulated or (**B**) up-regulated in response to 3D COL1. Microarray data were expressed as fluorescence intensities. Dashed line represents the background fluorescence.
(TIF)

S10 Fig 3D COL1 decreased the expression of heterogeneous nuclear ribonucleoparticle (hnRNP) protein-coding genes. Control (CTRL) and MT1-MMP (MT1) expressing MCF-7 cells were cultured for 24, 48 and 72h on 2D plastic (Plastic) or within 3D COL1 (Col3D). RNA was extracted from each sample and gene expression values measured using the Illumina Human HT-12 BeadChip array. The 24 probes corresponding to HNRNP genes were displayed as a heat map based on unsupervised hierarchical clustering. Red colour indicates genes that were up-regulated and green colour indicates genes that were down-regulated. Black indicates genes whose expression is unchanged in 3D COL1 as compared to 2D Plastic. Hierarchical clustering was performed using Euclidian as distance measure and average linkage.
(TIF)

S11 Fig Hierarchical clustering of probes modulated by MT1-MMP. Control (CTRL) and MT1-MMP (MT1) expressing MCF-7 cells were cultured for 24, 48 and 72h on 2D plastic (Plastic) or within 3D COL1 (Col3D). RNA was extracted from each sample and gene expression values measured using the Illumina Human HT-12 BeadChip array. (**A**) Heat map representation of normalized signal intensity values (log2) for probes altered by ≥ 1.8-fold in response to MT1-MMP expression. Red represents relative expression greater than the median expression level across all samples, and blue represents an expression level lower than the median expression level. The colour intensity represents the magnitude of the deviation from the median. The dendrogram at the left provides a measure of the relatedness of the probe expression profile in each sample. Hierarchical clustering was performed using Euclidian as distance measure and average linkage. (**B**) Number of probes modulated in response to the expression of MT1-MMP in MCF-7 cells growing on 2D plastic and 3D COL1.
(TIF)

S12 Fig MT1-MMP-dependent up-regulation of HIF target genes in MCF-7 cells included in 3D COL1. Microarray data were expressed as fluorescence intensities. Dashed line represents the background fluorescence.
(TIF)

S13 Fig 3D COL1-induction of BIK and ITGB1 in MCF-7 and ZR-75–1 cells. CTRL MCF-7 and ZR-75–1 cells were detached from plastic plates (Pellet) and cultured from 0.5 to 48 hours on 2D plastic (Plastic) or within 3D COL1 (COL3D). BIK and ITGB1 mRNA levels were quantified by semi-quantitative RT-PCR. Relative expression levels were obtained after normalization for the 28S rRNA levels. Data are means ± SEM (n = 3). * p<0.05, ** p<0.01, *** p<0.001 COL3D *versus* Plastic (one-way ANOVA with Bonferroni post tests).
(TIF)

S14 Fig Kinetic of filipodium-like cytoplasmic extensions protruding from CTRL MCF-7 cells embedded in 3D COL1 gel. CTRL MCF-7 cells embedded in 3D COL1 were imaged at 5 min intervals using a 40x Differential Interference Contrast objective. The arrow points to a vesicle left behind after the retraction of a cytoplasmic protrusion. Bar, 50 μm.
(TIF)

S15 Fig Paxillin localisation in CTRL cells embedded within 3D COL1. Representative confocal Z-series sections through a control MCF-7 cell embedded in 3D COL1. Paxillin (green), F-actin (red) and DRAQ5 (blue). *Inset*: higher magnification of vesicle displaying a colocalisation of actin and paxillin (yellow spots). *Arrows*, cytoplasmic protrusions displaying paxillin positivity; *arrowheads*, actin-rich vesicles displaying paxillin positivity. Bar, 10 μm.
(TIF)

S16 Fig 3D COL1 regulates DDR1 activation and cleavage. ZR-75–1 cells were cultured for 24h on 2D plastic (Plastic) or within 3D COL1 (Col3D) in the presence (+) of DDR1-IN-1 (1 μM) or vehicle (DMSO 0.1%). 3D COL1 gels were mechanically disrupted and lysed in RIPA buffer. Lysates were resolved by reducing 8% SDS-PAGE followed by immunoblot analysis. Blots were probed with phospho-DDR1 (Tyr792) antibody (*A*) and then reprobed with antibodies directed against the cytosolic juxtamembrane domain of DDR1 (*B*), BIK (*C*) or **β**-actin (*D*), as a loading control. *Black arrow*, phospho-DDR1; *, non-specific immuno-reactive band. Lysates of 3T3 cells transfected with human DDR1b cDNA [101], which contains both 120-kDa full-length and 62-kDa C-terminal DDR1 species were included as a positive control.
(TIF)

S1 Movie Behaviour of CTRL cells included in 3D COL1 gel. Time-lapse video of control MCF-7 cells (CTRL) embedded in a 3D COL1 gel for 16 hours. Images were acquired every 10 minutes with a Nikon A1R time-lapse microscope equipped with a microscope stage incubation chamber (atmosphere of 5% CO_2_ at 37°C) and a S Plan Fluor ELWD 20x DIC N1 objective.
(ZIP)

S2 Movie Behaviour of MT1 MCF-7 cells included in 3D COL1 gel. Time-lapse video of MT1-MMP expressing MCF-7 cells (MT1) embedded in a 3D COL1 gel for 16 hours. Images were acquired every 10 minutes with a Nikon A1R time-lapse microscope equipped with a microscope stage incubation chamber (atmosphere of 5% CO_2_ at 37°C) and a S Plan Fluor ELWD 20x DIC N1 objective.
(ZIP)

S3 Movie Influence of BB-94, a synthetic MMP inhibitor, on the behaviour of MT1 MCF-7 cells included in 3D COL1 gel. Time-lapse video of MT1-MMP expressing MCF-7 cells (MT1) embedded in a 3D COL1 gel for 16 hours in the presence of BB-94 (1 μM). Images were acquired every 10 minutes with a Nikon A1R time-lapse microscope equipped with a microscope stage incubation chamber (atmosphere of 5% CO_2_ at 37°C) and a S Plan Fluor ELWD 20x DIC N1 objective.
(ZIP)

S4 Movie Influence of BB-94, a synthetic MMP inhibitor, on the behaviour of CTRL cells included in 3D COL1 gel. Time-lapse video of control MCF-7 cells (CTRL) embedded in a 3D COL1 gel for 16 hours in the presence of BB-94 (1 μM). Images were acquired every 10 minutes with a Nikon A1R time-lapse microscope equipped with a microscope stage incubation chamber (atmosphere of 5% CO_2_ at 37°C) and a S Plan Fluor ELWD 20x DIC N1 objective.
(ZIP)

S5 Movie Behaviour of DMSO-treated MCF-7 cells included in 3D COL1 gel. Time-lapse video of DMSO-treated MCF-7 cells embedded in a 3D COL1 gel for 16 hours. Images were acquired every 10 minutes with a Nikon A1R time-lapse microscope equipped with a microscope stage incubation chamber (atmosphere of 5% CO_2_ at 37°C) and a S Plan Fluor ELWD 20x DIC N1 objective.
(AVI)

S6 Movie Behaviour of PP2-treated MCF-7 cells included in 3D COL1 gel. Time-lapse video of PP2-treated MCF-7 cells embedded in a 3D COL1 gel for 16 hours. Images were acquired every 10 minutes with a Nikon A1R time-lapse microscope equipped with a microscope stage incubation chamber (atmosphere of 5% CO_2_ at 37°C) and a S Plan Fluor ELWD 20x DIC N1 objective.
(AVI)

S1 Supplementary Methods Supporting Information Text. (DOCX)

S1 Table Sequences of RT-PCR primers used. (DOCX)

S2 Table Full GO Term analysis of individual sublists. (XLSX)

S3 Table 3D COL1 core signature. List of genes similarly modulated in CTRL and MT1 MCF-7 cells in response to 3D COL1.
(XLSX)

S4 Table Statistical analysis of FACS analysis data (S6 Fig.). (XLSX)

S5 Table List of genes consistently modulated by MT1-MMP in MCF-7 cells plated on 2D plastic during 24, 48 and 72 hours. (DOCX)

S6 Table List of genes modulated by MT1-MMP in MCF-7 cells embedded within 3D COL1. (DOCX)

S7 Table Bio-functions associated with the “3D COL1 core signature”. (XLSX)

S8 Table Gene ontology terms associated with the 3D COL1 signature. (XLSX)
