# Supplementary figures and images for: A Membrane-Type-1 Matrix Metalloproteinase (MT1-MMP) – Discoidin Domain Receptor 1 Axis Regulates Collagen-Induced Apoptosis in Breast Cancer Cells
Source: PLoS One. 2015 Mar 16;10(3):e0116006. doi: 10.1371/journal.pone.0116006 (PMC4638154; doi:10.1371/journal.pone.0116006)

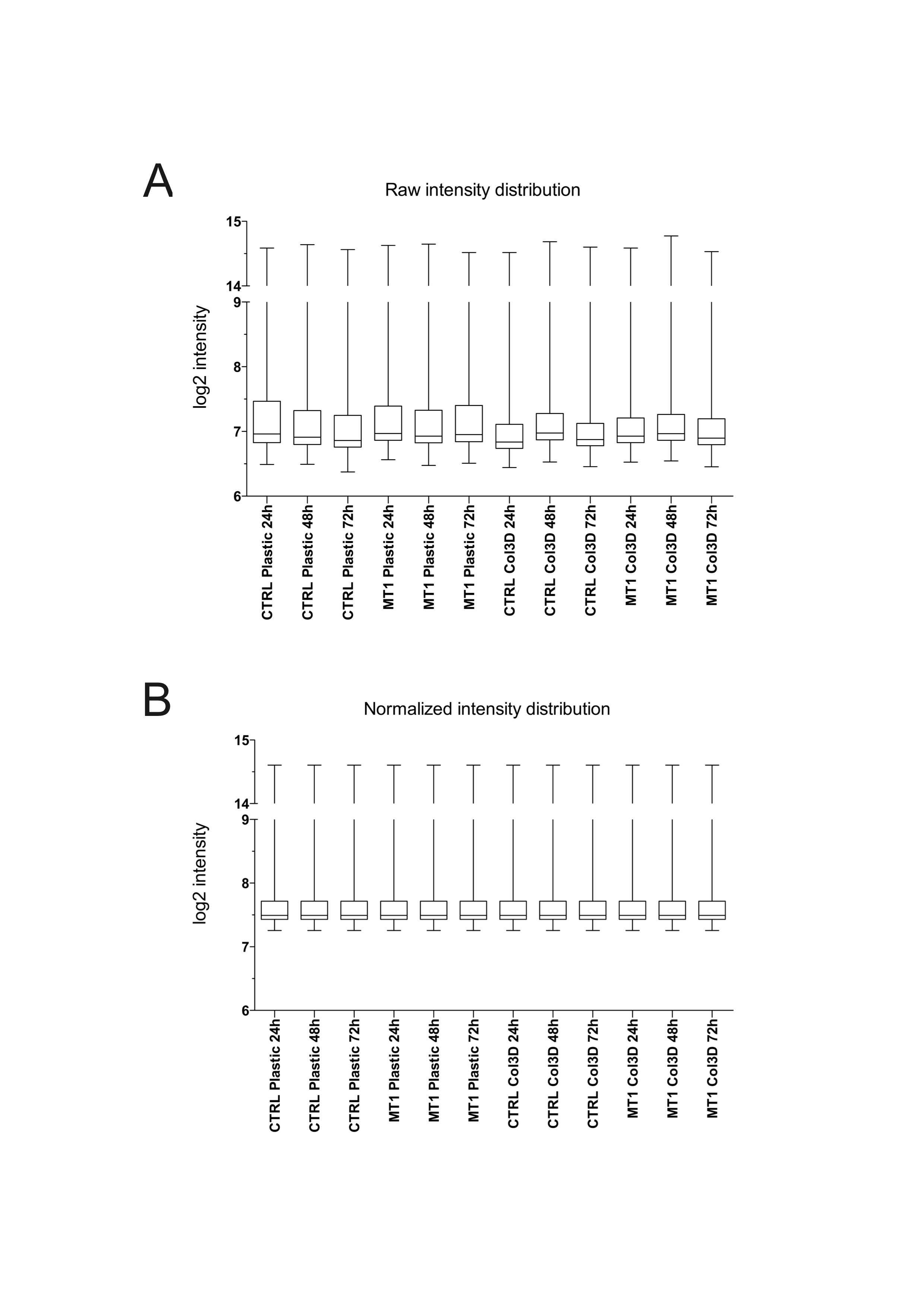

Supplement: S1 Fig — Box plot depicting the difference among microarray expression datasets from each sample before (A) and after (B) normalization by variance-stabilizing transformation and quantile normalisation performed using the R package lumi in Chipster analysis software. Boxes show the 25th and 75th percentiles in the distribution of log-transformed (log base 2) intensities. The median is the horizontal bar in the middle of the box. (TIF) [file pone.0116006.s001.tif]

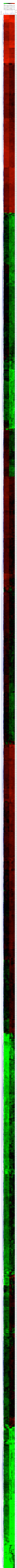

Supplement: S2 Fig — Red represents relative expression greater than the median expression level across all samples, and blue represents an expression level lower than the median expression level. The colour intensity represents the magnitude of the deviation from the median. The dendrogram at the left provides a measure of the relatedness of the probe expression profile in each sample. The dendrogram at the top provides a measure of the relatedness of the 12 samples. (PDF) [file pone.0116006.s002.pdf]

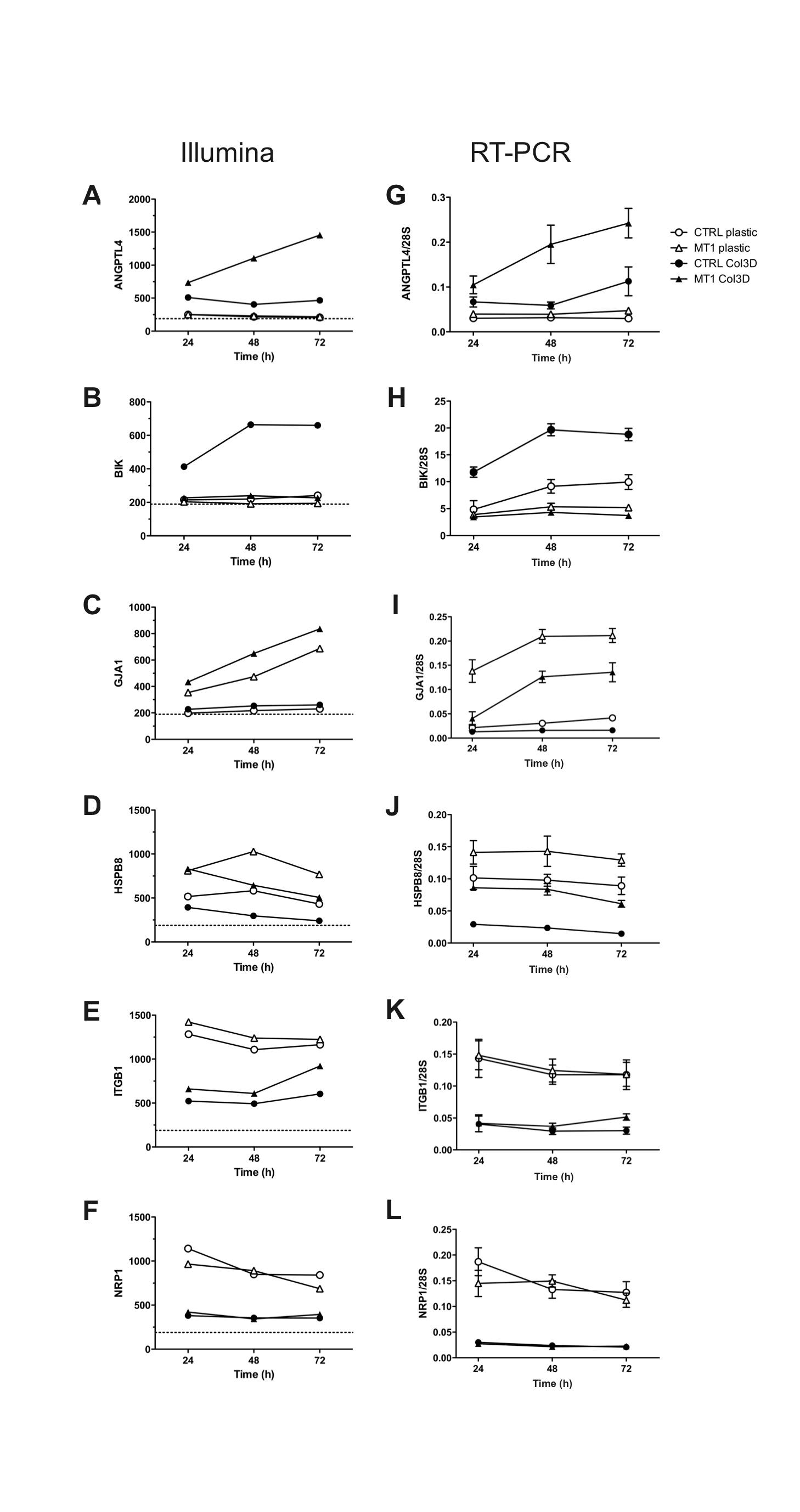

Supplement: S3 Fig — Six differentially expressed genes revealed by microarray analysis (A-F) were validated by semi-quantitative RT-PCR (G-L). Microarray data were expressed as fluorescence intensities. Dashed line represents the background fluorescence. For semi-quantitative RT-PCR, relative expression levels were obtained after normalization for the 28S rRNA levels. Data are means ± SEM (n = 4). (TIF) [file pone.0116006.s003.tif]

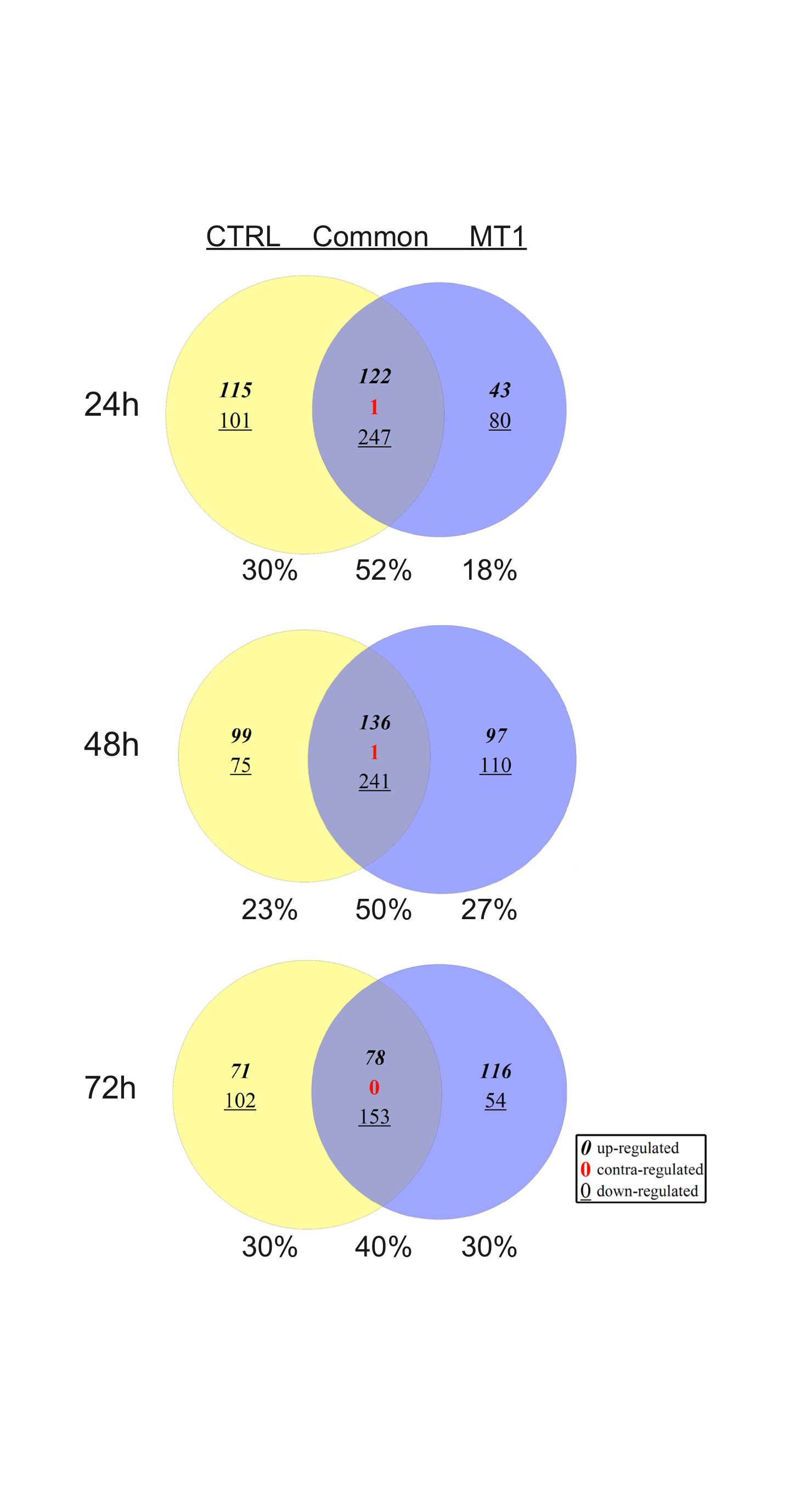

Supplement: S4 Fig — Numbers in italics, red, and underlined represent up-, contra-, and down-regulated genes, respectively. Numbers in brackets refer to the numbers of genes modulated at each time point. Percentages represent the proportion of genes present in each area of the diagrams. (TIF) [file pone.0116006.s004.tif]

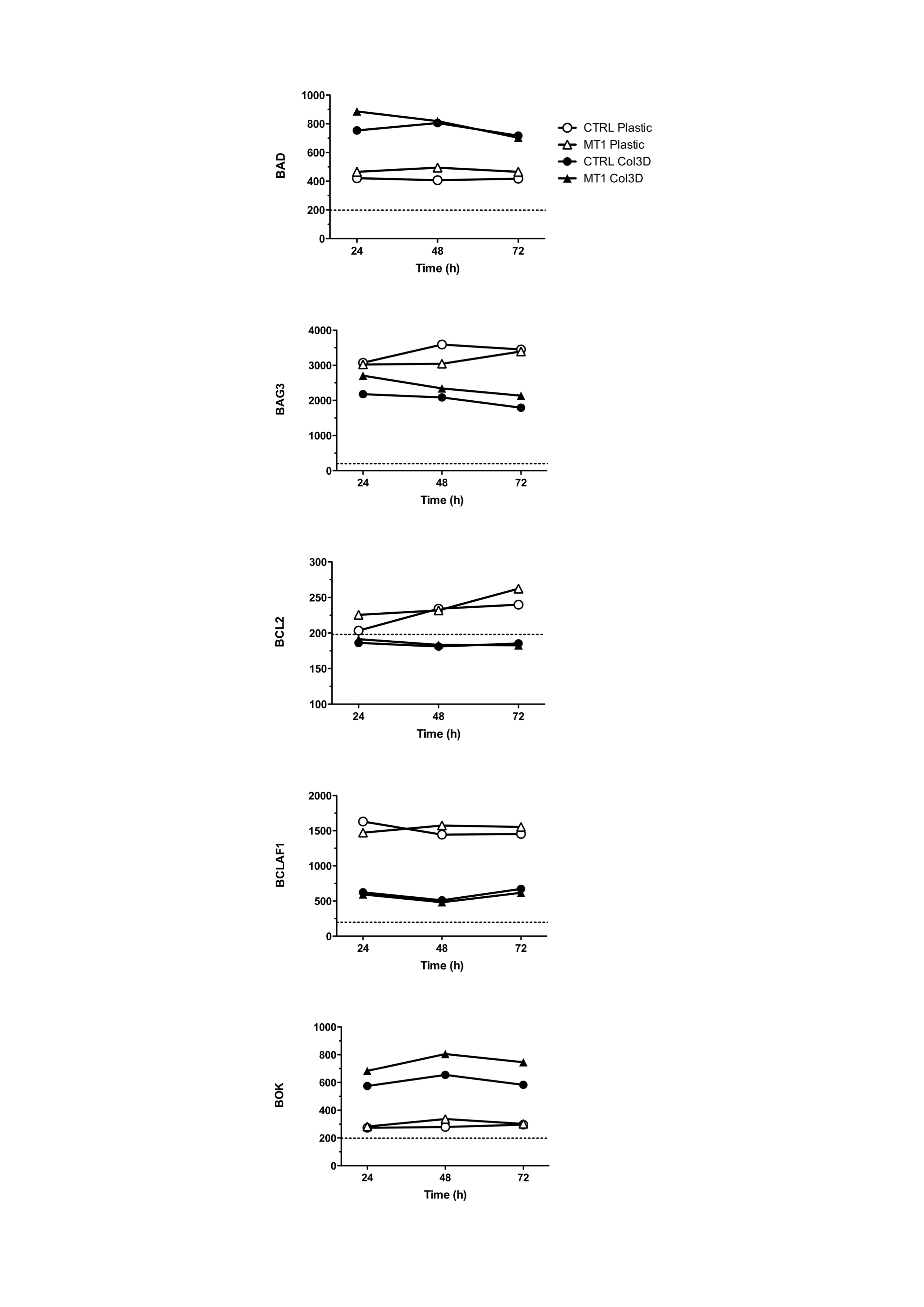

Supplement: S5 Fig — Microarray data were expressed as fluorescence intensities. Dashed line represents the background fluorescence. (TIF) [file pone.0116006.s005.tif]

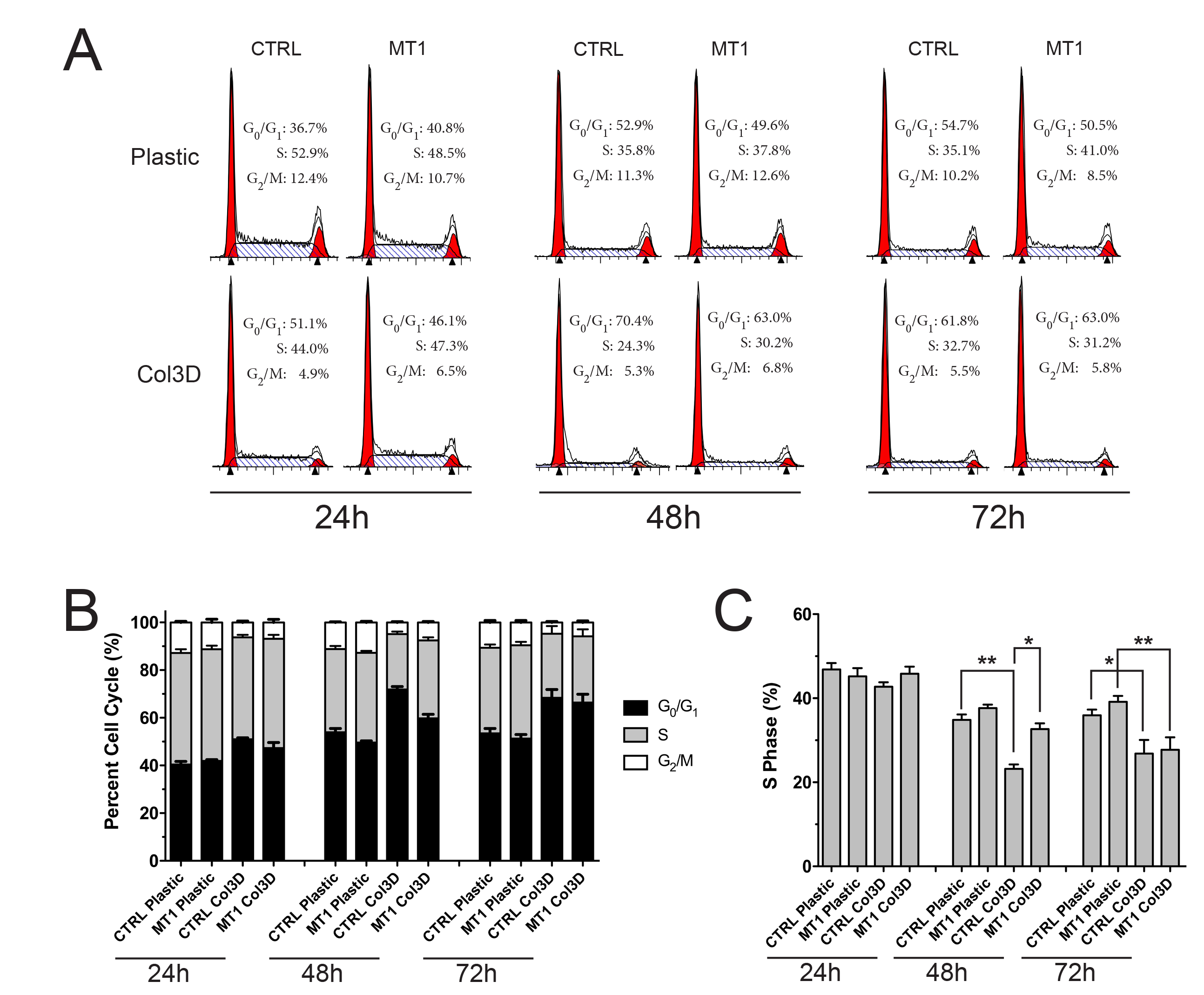

Supplement: S6 Fig — For fluorescence-activated cell sorting (FACS) analysis, control (CTRL) and MT1-MMP expressing (MT1) MCF-7 cells were cultured during 24, 48 and 72h on Plastic or within 3D COL1. Nuclei were isolated and stained with propidium iodide buffer followed by cell sorting analysis. (A) The acquired FACS data were analysed by ModFit LT software. (B) The results of FACS analysis are presented as mean (±SEM) for four independent experiments. The detailed statistical analysis for each group is illustrated in S4 Table. (C) The percentage of cells in S phase is shown. Data are means ± SEM (n = 4). * p<0.05, *** p<0.001 MT1 versus CTRL; # p<0.05, ### p<0.001 Col3D versus Plastic (two-way ANOVA with Bonferroni post tests); *, genotype effect; #, matrix effect). (TIF) [file pone.0116006.s006.tif]

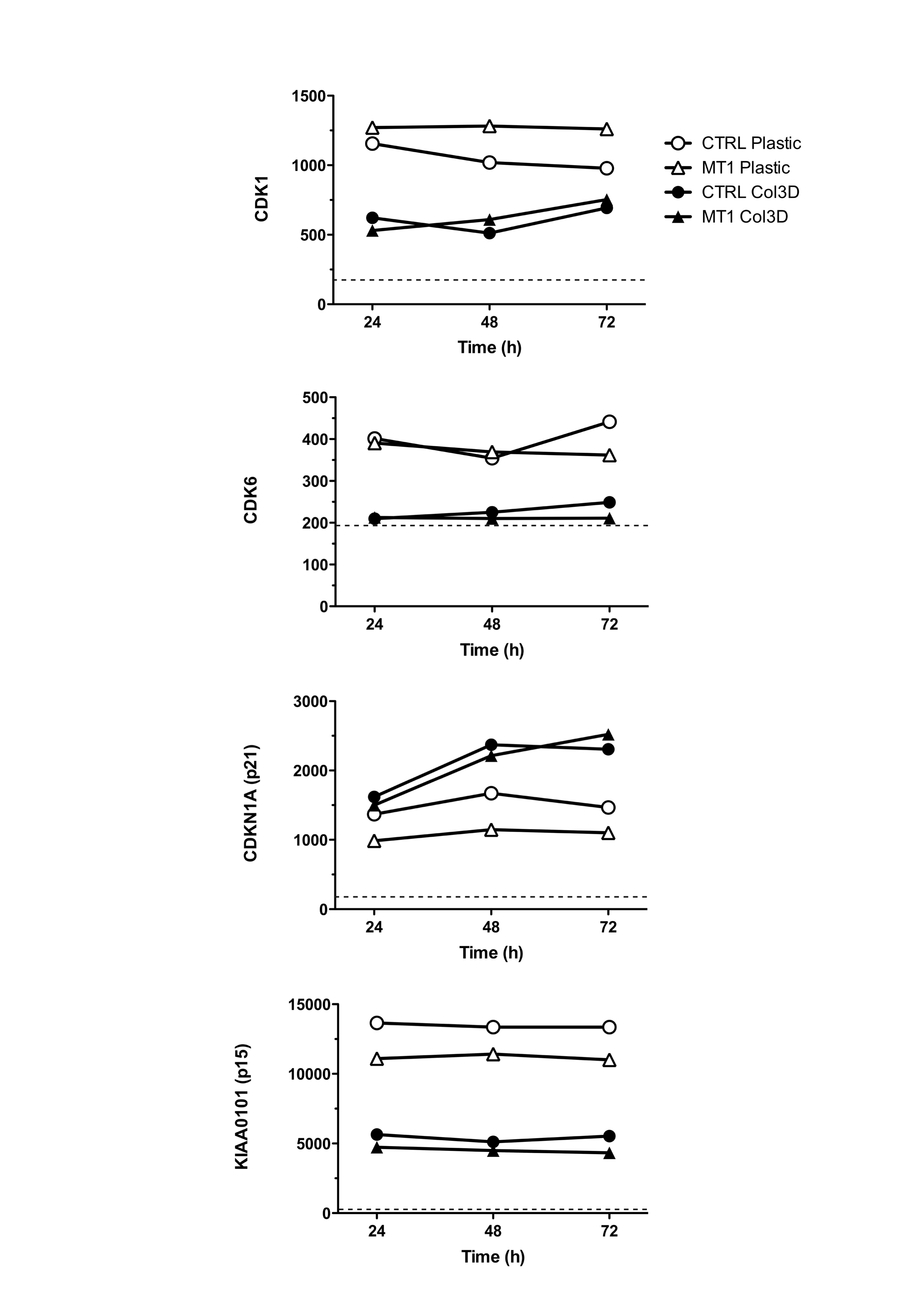

Supplement: S7 Fig — Microarray data were expressed as fluorescence intensities. Dashed line represents the background fluorescence. (TIF) [file pone.0116006.s007.tif]

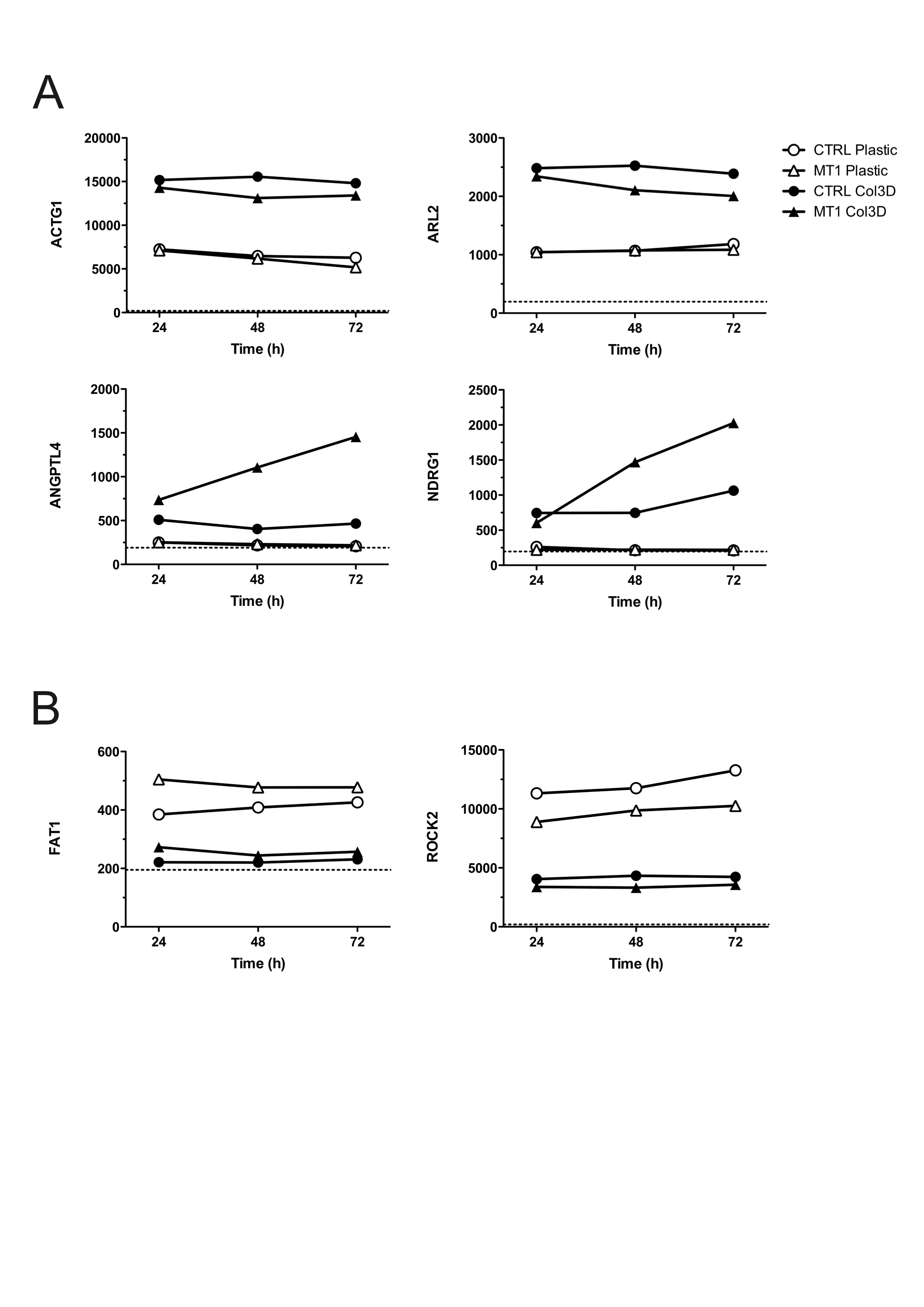

Supplement: S8 Fig — Microarray data were expressed as fluorescence intensities. Dashed line represents the background fluorescence. (TIF) [file pone.0116006.s008.tif]

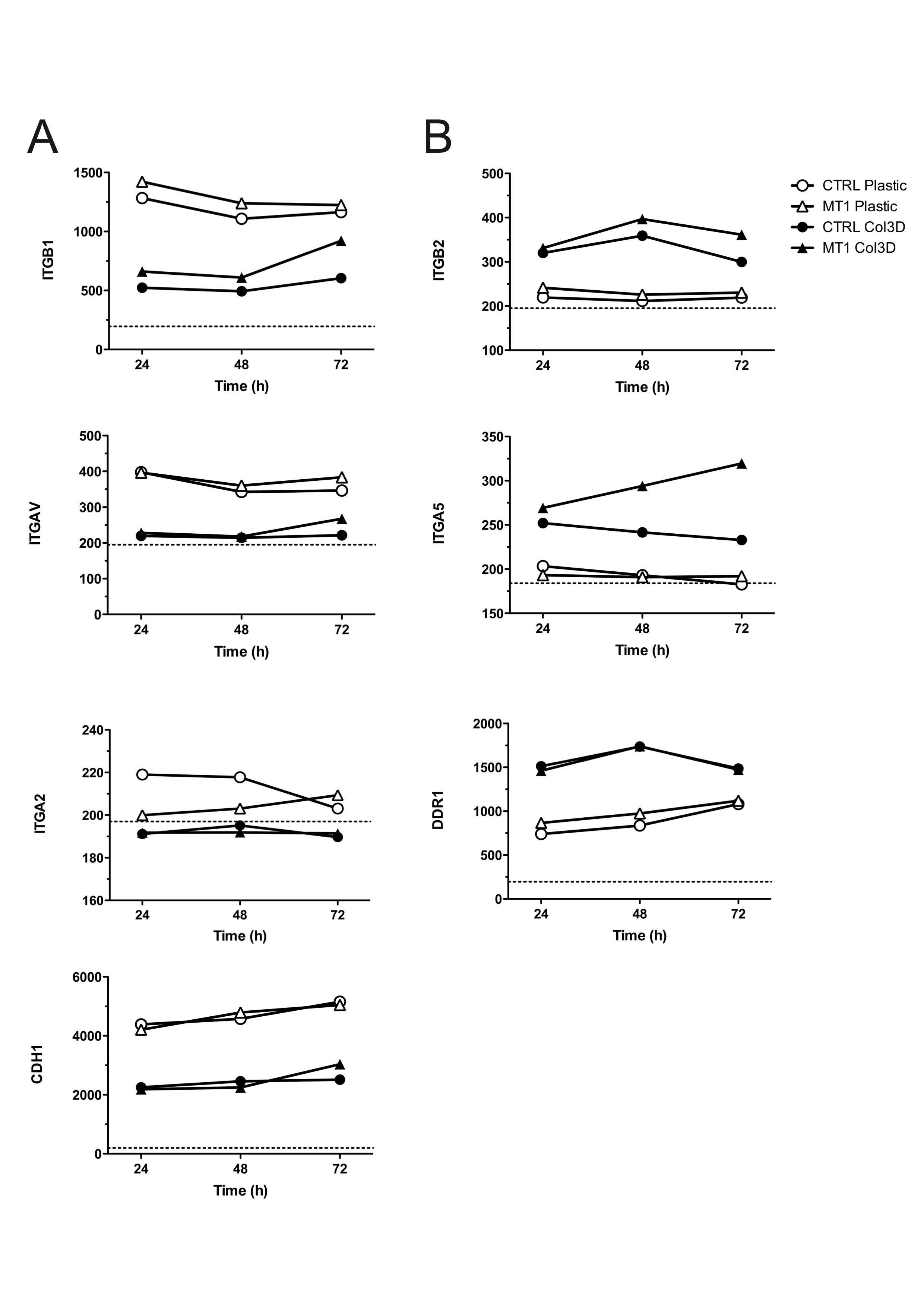

Supplement: S9 Fig — The genes were (A) down-regulated or (B) up-regulated in response to 3D COL1. Microarray data were expressed as fluorescence intensities. Dashed line represents the background fluorescence. (TIF) [file pone.0116006.s009.tif]

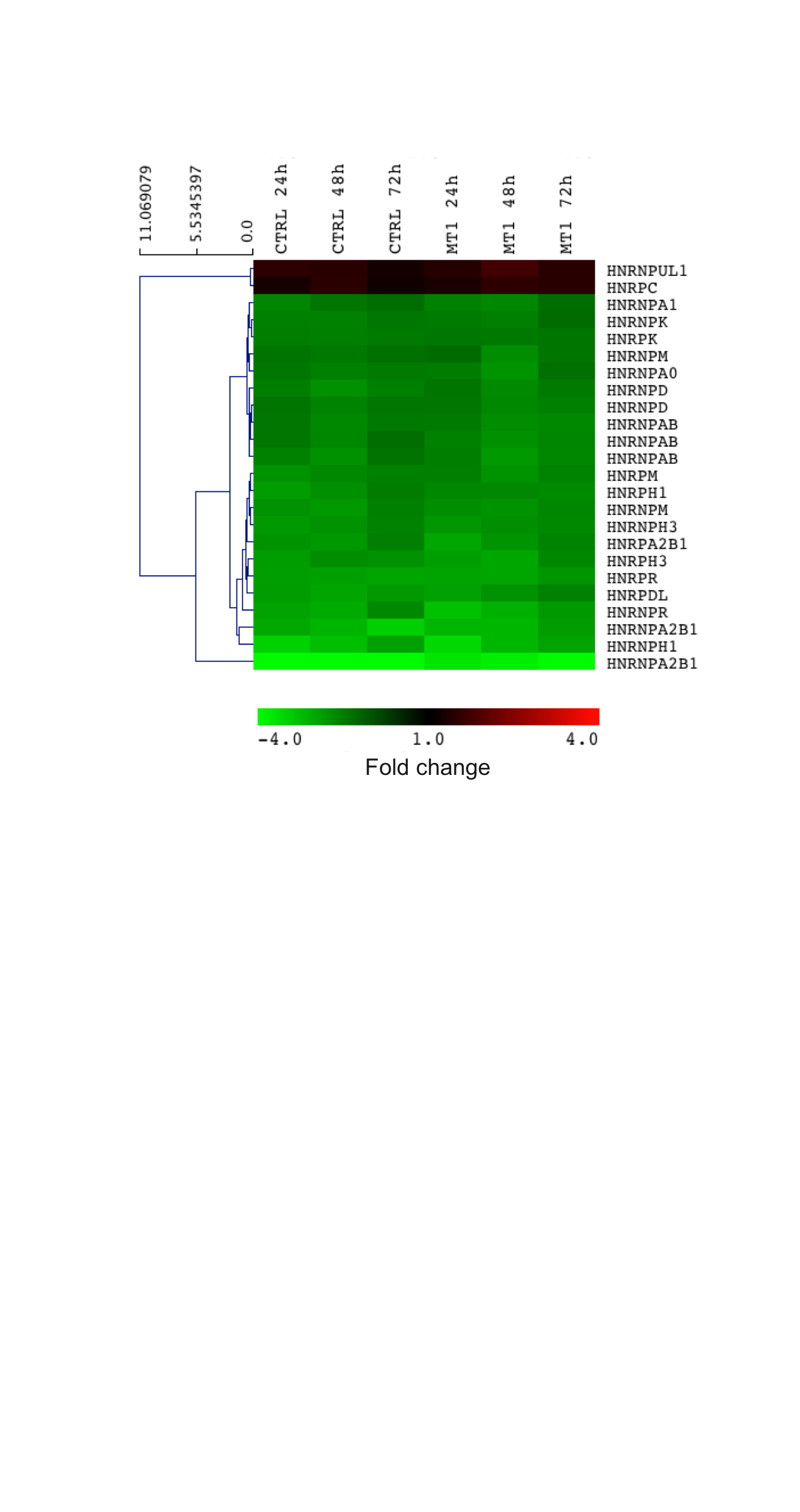

Supplement: S10 Fig — Control (CTRL) and MT1-MMP (MT1) expressing MCF-7 cells were cultured for 24, 48 and 72h on 2D plastic (Plastic) or within 3D COL1 (Col3D). RNA was extracted from each sample and gene expression values measured using the Illumina Human HT-12 BeadChip array. The 24 probes corresponding to HNRNP genes were displayed as a heat map based on unsupervised hierarchical clustering. Red colour indicates genes that were up-regulated and green colour indicates genes that were down-regulated. Black indicates genes whose expression is unchanged in 3D COL1 as compared to 2D Plastic. Hierarchical clustering was performed using Euclidian as distance measure and average linkage. (TIF) [file pone.0116006.s010.tif]

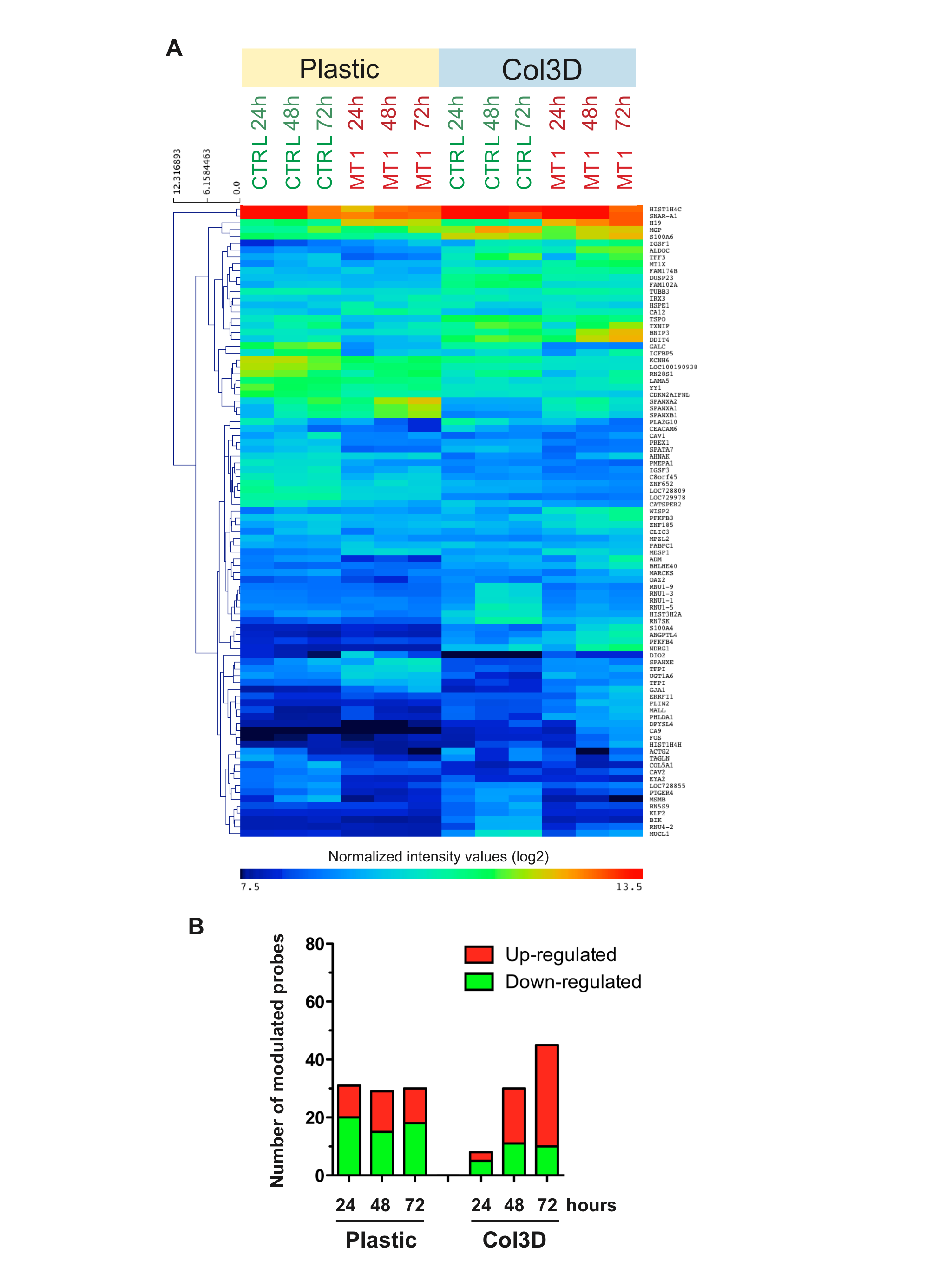

Supplement: S11 Fig — Control (CTRL) and MT1-MMP (MT1) expressing MCF-7 cells were cultured for 24, 48 and 72h on 2D plastic (Plastic) or within 3D COL1 (Col3D). RNA was extracted from each sample and gene expression values measured using the Illumina Human HT-12 BeadChip array. (A) Heat map representation of normalized signal intensity values (log2) for probes altered by ≥ 1.8-fold in response to MT1-MMP expression. Red represents relative expression greater than the median expression level across all samples, and blue represents an expression level lower than the median expression level. The colour intensity represents the magnitude of the deviation from the median. The dendrogram at the left provides a measure of the relatedness of the probe expression profile in each sample. Hierarchical clustering was performed using Euclidian as distance measure and average linkage. (B) Number of probes modulated in response to the expression of MT1-MMP in MCF-7 cells growing on 2D plastic and 3D COL1. (TIF) [file pone.0116006.s011.tif]

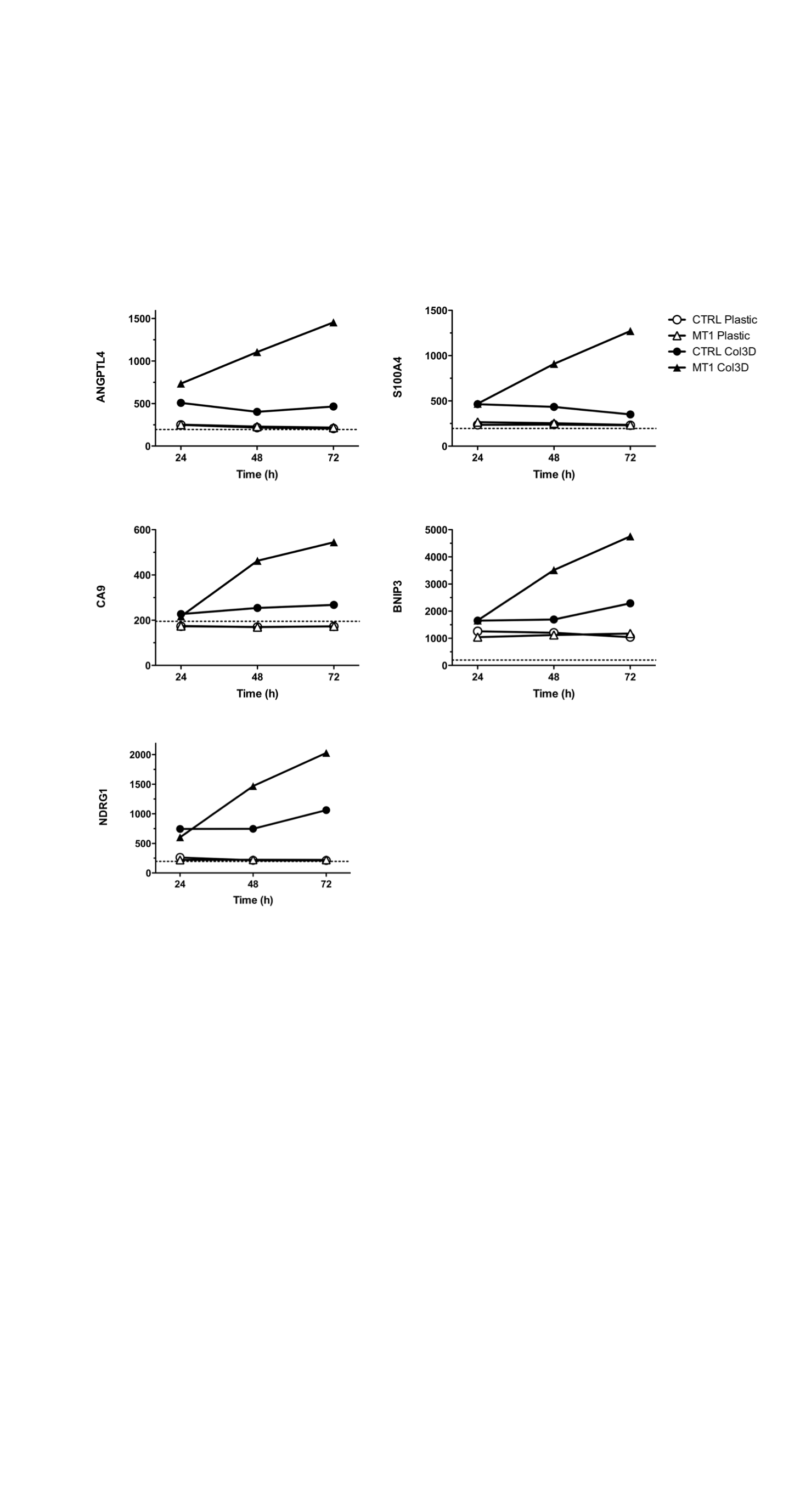

Supplement: S12 Fig — Microarray data were expressed as fluorescence intensities. Dashed line represents the background fluorescence. (TIF) [file pone.0116006.s012.tif]

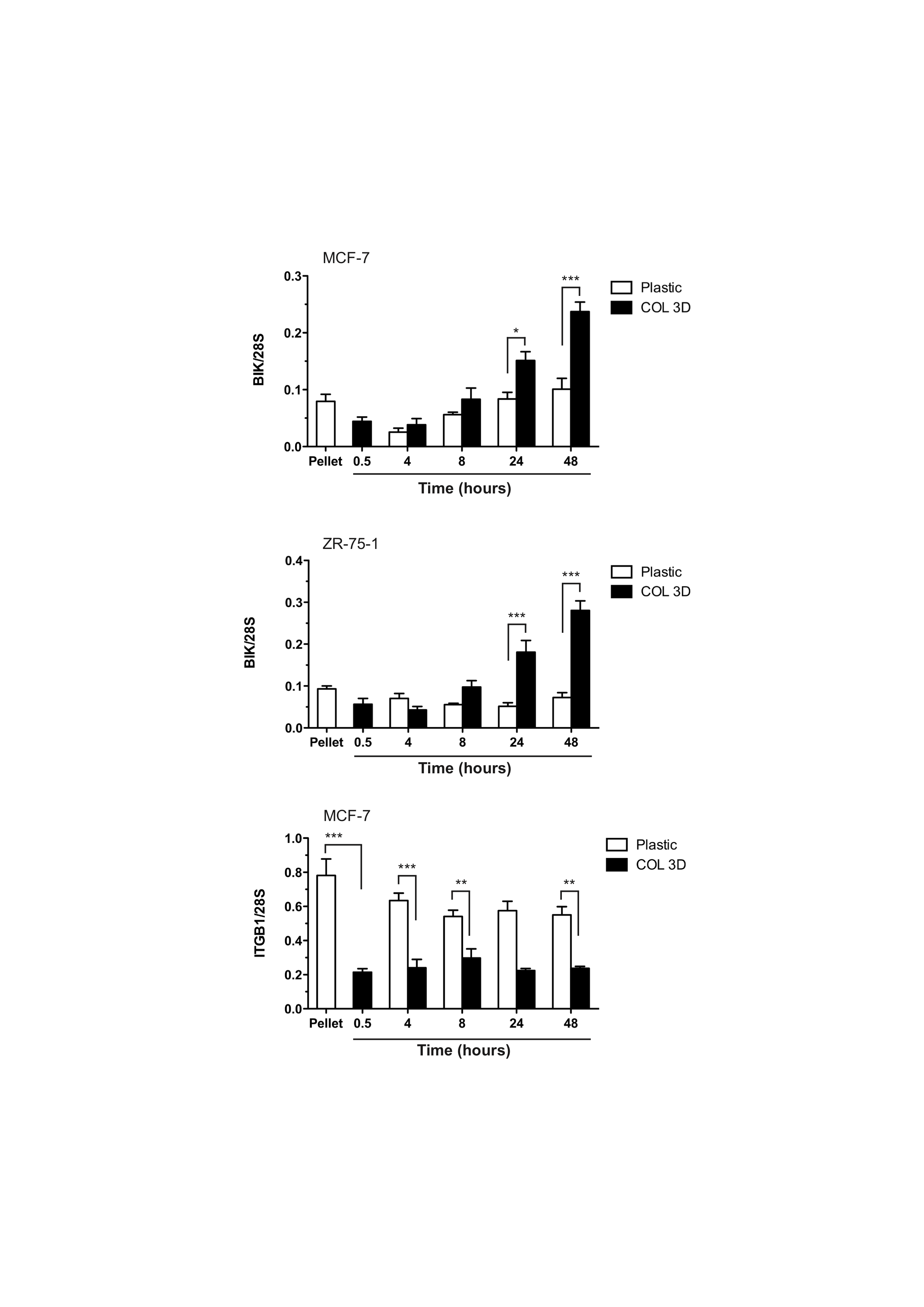

Supplement: S13 Fig — CTRL MCF-7 and ZR-75–1 cells were detached from plastic plates (Pellet) and cultured from 0.5 to 48 hours on 2D plastic (Plastic) or within 3D COL1 (COL3D). BIK and ITGB1 mRNA levels were quantified by semi-quantitative RT-PCR. Relative expression levels were obtained after normalization for the 28S rRNA levels. Data are means ± SEM (n = 3). * p<0.05, ** p<0.01, *** p<0.001 COL3D versus Plastic (one-way ANOVA with Bonferroni post tests). (TIF) [file pone.0116006.s013.tif]

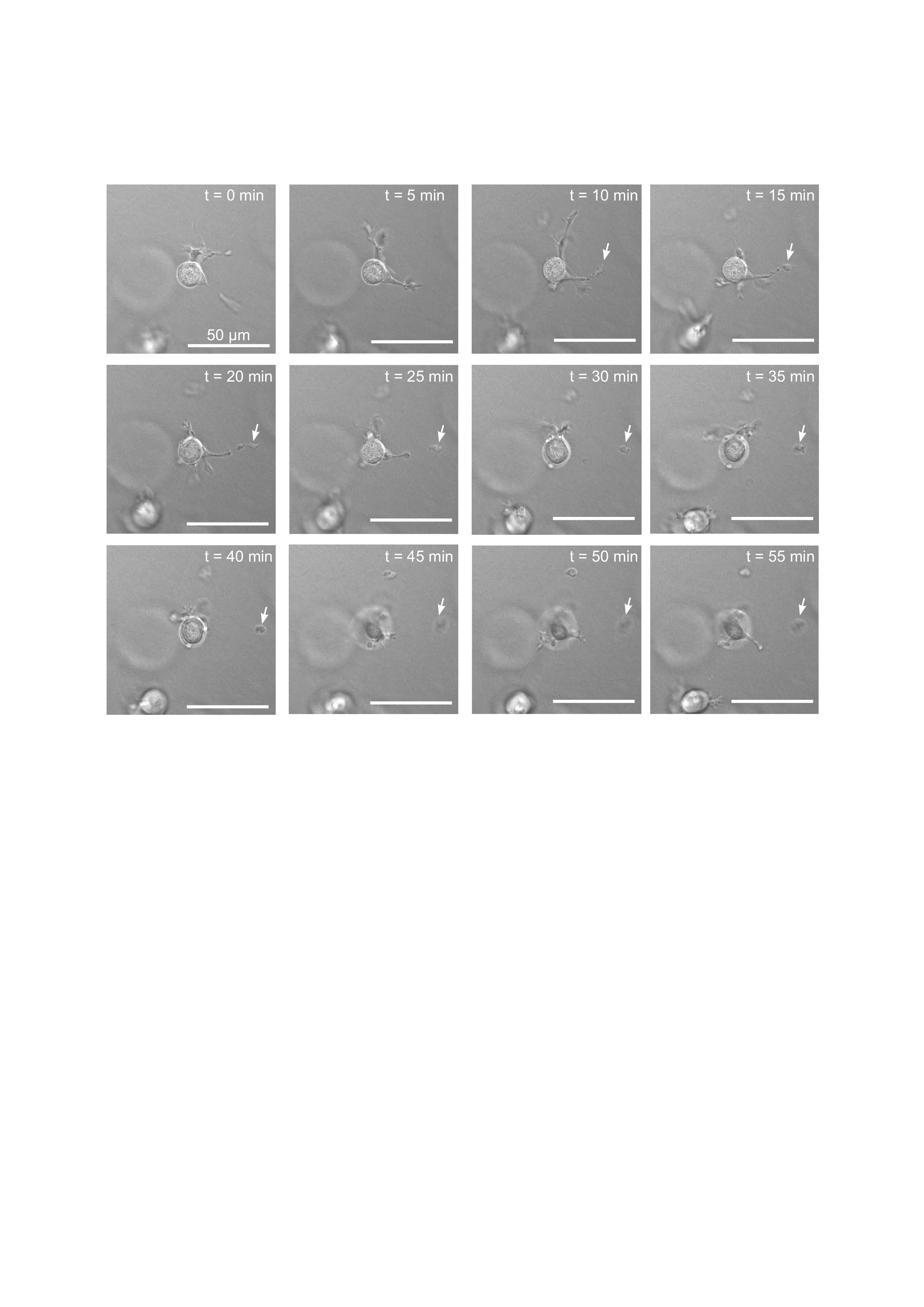

Supplement: S14 Fig — CTRL MCF-7 cells embedded in 3D COL1 were imaged at 5 min intervals using a 40x Differential Interference Contrast objective. The arrow points to a vesicle left behind after the retraction of a cytoplasmic protrusion. Bar, 50 μm. (TIF) [file pone.0116006.s014.tif]

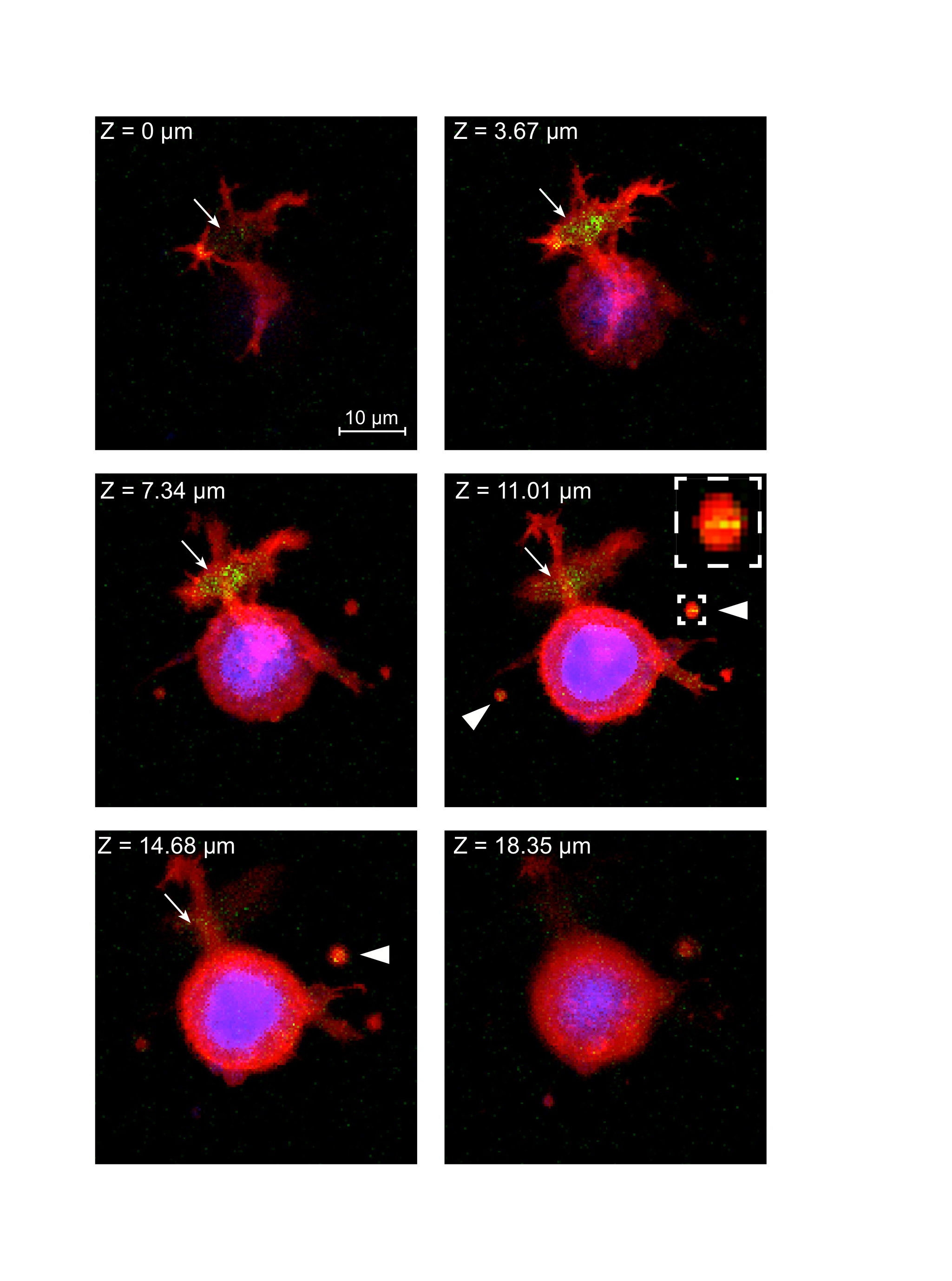

Supplement: S15 Fig — Representative confocal Z-series sections through a control MCF-7 cell embedded in 3D COL1. Paxillin (green), F-actin (red) and DRAQ5 (blue). Inset: higher magnification of vesicle displaying a colocalisation of actin and paxillin (yellow spots). Arrows, cytoplasmic protrusions displaying paxillin positivity; arrowheads, actin-rich vesicles displaying paxillin positivity. Bar, 10 μm. (TIF) [file pone.0116006.s015.tif]

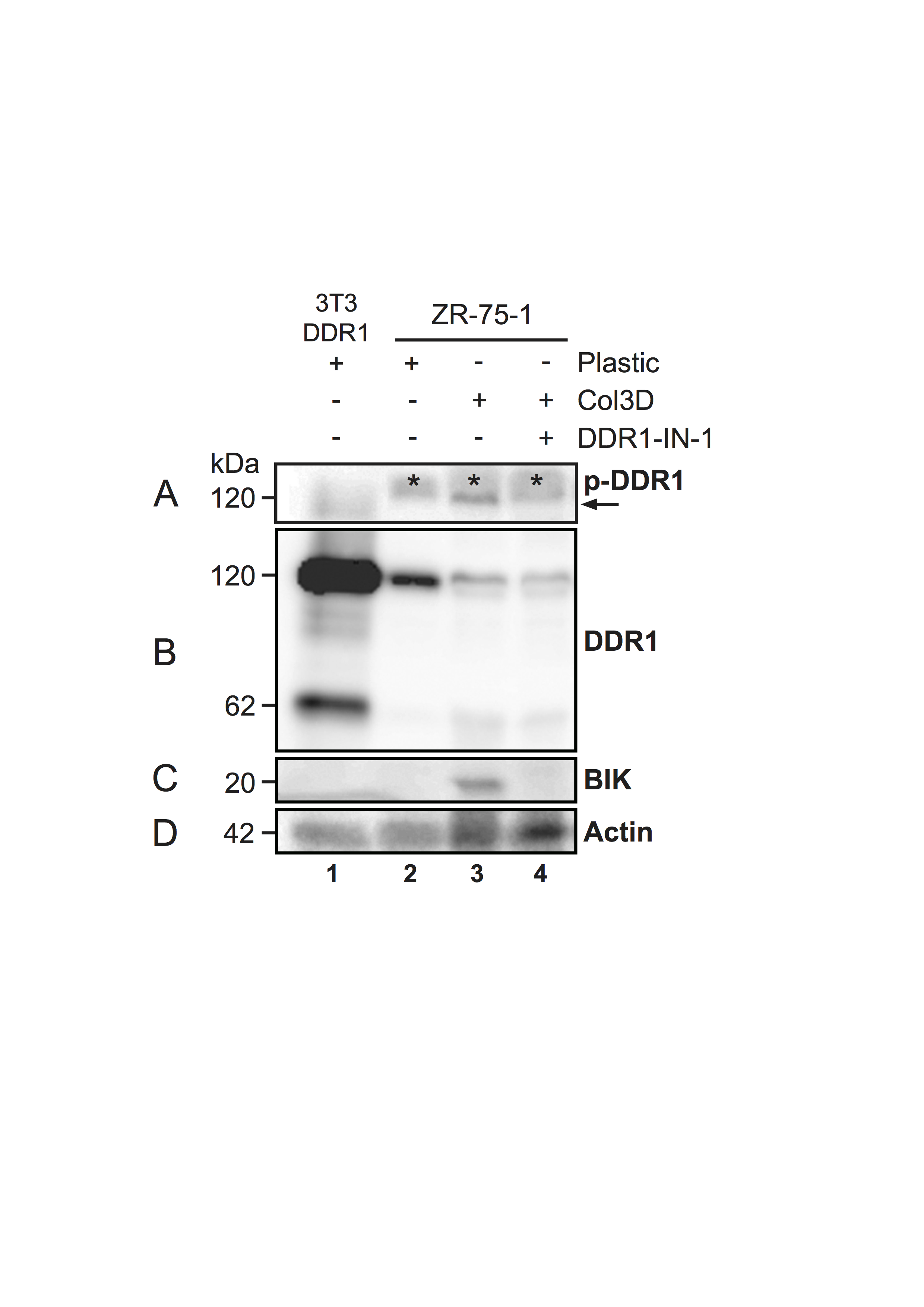

Supplement: S16 Fig — ZR-75–1 cells were cultured for 24h on 2D plastic (Plastic) or within 3D COL1 (Col3D) in the presence (+) of DDR1-IN-1 (1 μM) or vehicle (DMSO 0.1%). 3D COL1 gels were mechanically disrupted and lysed in RIPA buffer. Lysates were resolved by reducing 8% SDS-PAGE followed by immunoblot analysis. Blots were probed with phospho-DDR1 (Tyr792) antibody (A) and then reprobed with antibodies directed against the cytosolic juxtamembrane domain of DDR1 (B), BIK (C) or β-actin (D), as a loading control. Black arrow, phospho-DDR1; *, non-specific immuno-reactive band. Lysates of 3T3 cells transfected with human DDR1b cDNA [101], which contains both 120-kDa full-length and 62-kDa C-terminal DDR1 species were included as a positive control. (TIF) [file pone.0116006.s016.tif]
