# Supplementary material for: A Membrane-Type-1 Matrix Metalloproteinase (MT1-MMP) – Discoidin Domain Receptor 1 Axis Regulates Collagen-Induced Apoptosis in Breast Cancer Cells
Source: PLoS One. 2015 Mar 16;10(3):e0116006. doi: 10.1371/journal.pone.0116006 (PMC4638154; doi:10.1371/journal.pone.0116006)
Supplement: S1 Supplementary Methods — (DOCX) [file pone.0116006.s023.docx]

Supplementary methods section for

**“A membrane-type-1 matrix metalloproteinase (MT1-MMP) – discoidin domain receptor 1 axis regulates collagen-induced apoptosis in breast cancer cells”**

Delphine Assent^1^, Isabelle Bourgot^1^, Benoît Hennuy^2^, Pierre Geurts^3^, Agnès Noël^1^, Jean-Michel Foidart^1^, Erik Maquoi^1,^*

^1^ Laboratory of Tumour and Developmental Biology, Groupe Interdisciplinaire de Génoprotéomique Appliqué (GIGA), Unit of Cancer, University of Liège, Liège, Belgium

^2^ GIGA-Genomics platform, University of Liège, Liège, Belgium

^3^ Systems and Modelling, Department of Electrical Engineering and Computer Science & GIGA-R, University of Liège, Liège, Belgium

* E-mail: erik.maquoi@ulg.ac.be

Supplementary Methods

RNA interference

Endoribonuclease-prepared siRNAs (esiRNAs) targeting human DDR1 and EGFP (negative control) were purchased from Sigma-Aldrich (St-Louis, MO, USA). For transfection of esiRNAs, 250,000 MCF-7 cells were plated in six-well plates in 2 ml/well of culture medium. Once adherent, esiRNAs (1 µg/well) were transfected by using phosphate calcium precipitation as described previously (Bindels *et al*., 2006). Transfections were repeated after 24h. 24h after the second transfection, the cells were extensively washed with PBS, collected, seeded within COL1 gels and cultured for 2 days.

Western blot analysis

Total cell extracts were prepared by incubating cells pellets in radioimmune precipitation assay (RIPA) buffer as described previously (Maquoi *et al*., 2000). Samples of total cell extracts (30 µg) were resolved by SDS-PAGE under reducing conditions, and proteins were transferred to polyvinylidene difluoride membranes (PerkinElmer, Zaventem, Belgium). The membranes were exposed to antibodies used at concentrations recommended by the manufacturers. BIK-specific (N-19) antibody was from Santa Cruz Biotechnology, phospho-DDR1 (Tyr792) (#11994) and total DDR1 (#5583) antibodies were from Cell Signaling Technology (Danvers, MA, USA), β-actin-specific antibody was from Sigma (St. Louis, MO, USA). Protein loading was controlled by β-actin immunodetection. Immunocomplexes were visualized by chemiluminescence reaction on a luminescent image analyzer (LAS-4000). Intensity of bands was quantified using Quantity-One software (Bio-Rad Laboratories, Nazareth Eke, Belgium) and normalized with respect to β-actin expression.

Immunofluorescence microscopy

Cells were cultured in µ-slide Angiogenesis (Ibidi) at a density of 4 x 10^3^ cells/well or within 3D COL1 gels (14 x 10^3^ cells/well). Cells were treated with 1 µM Saracatinib (a c-Src/Abl kinase inhibitor), vehicle (DMSO 0.1%), or left untreated for 48 hours. After treatment, cells were washed in PBS, fixed using 4% paraformaldehyde in PBS for 30 mins, permeabilized in 0.1% Triton X-100, and blocked with normal goat serum (1/20 dilution in PBS). Cells were incubated with mouse monoclonal anti-paxillin antibody (1/500 dilution in PBS, DB Transduction Laboratories) for 2 hrs at room temperature, rinsed with PBS, and subsequently incubated with goat anti-mouse antibody labelled with Alexa 488 (Invitrogen) for 1 hr. After washing the cells with PBS, F-actin and nuclei were labelled with phalloidin-Atto550 (1/500 dilution in PBS, Sigma) and DRAQ5 (1/1000 dilution in PBS, Biostatus, Shepshed, United Kingdom) for 20 mins at room temperature. After washings, images were acquired in each of three channels (525 nm, 595 nm and 700 nm) by laser scanning confocal microscopy with sequential Z-stage scanning (Nikon A1R confocal microscope with a S Plan Fluor ELWD 40x DIC N1 objective).

Time-lapse imaging

For time-lapse microscopy imaging of cells growing in 3D COL1, cells were suspended in a neutralized solution of native acid-extracted rat tail COL1 (2 mg/ml) at a density of 15 x 10^5^ cells/ml and the suspensions were seeded into µ-slide VI^0.4^ flow chambers (Ibidi, Martinsried, Germany) and polymerized at 37°C. Micro-slide chambers were then imaged for 1 hour at 5 min intervals or for 16 hours at 10 min intervals with a Nikon A1R time-lapse microscope equipped with a microscope stage incubation chamber (atmosphere of 5% CO_2_ at 37°C) and a S Plan Fluor ELWD 40x DIC N1 objective or a S Plan Fluor ELWD 20x DIC N1 objective, respectively.

Biological pathway analyses

Data set consisting of genes altered by ≥1.8-fold were uploaded into Ingenuity Pathway Analysis (IPA) system (Ingenuity^®^ System, [http://www.ingenuity.com](http://www.ingenuity.com/)). IPA was used to verify if differentially expressed genes were related more than expected by chance to networks, biofunctions and canonical pathways. IPA allows adding structure to the vast amount of data generated by microarrays. To begin, an input file containing fold changes of all probe sets was uploaded into IPA system. From this file, dataset in Core Analysis was produced. General settings for IPA system as «Ingenuity^®^ Knowledge Base (genes)» and «considered only molecules and/or relationships for humans» were used. IPA calculates a *p*-value based on the right-tailed Fisher’s exact test for each canonical pathway, which is a measure of the likelihood that the association of a data set with a pathway is due to random chance. A cutoff of 1.2 was set. Relevant pathways with *p*-values smaller than 0.05, were taken into account. IPA also allows to perform an «Upstream regulator Analysis» (URA). The goal of URA is to identify upstream regulators (ie., transcription factor, microRNA, kinase, compound…) explaining differentially expressed genes in the data, as well as their directions of changes [43]. Causal effects between upstream regulators and their target genes are derived from literature and compiled in the «Ingenuity^®^ Knowledge Base». For each upstream regulator, URA first computes an overlap *p*-value (using Fisher’s Exact Test), which measures whether the overlap between the target genes of this regulator and the differentially expressed genes is statistically significant. Then, URA compares the direction of changes of the differentially expressed genes with expectations based on the literature to predict for each upstream regulator an activation state (up- or down-regulated). This prediction is derived from an activation z-score that quantifies the consistency between the observed (in the data) and expected (in the knowledge base) directions of changes. A high absolute value of this z-score (≥ 2) indicates a statistically significant match between observed and expected differences. A positive z-score then corresponds to an activated or up-regulated regulator, a negative z-score to an inhibited or down-regulated regulator.

Gene Ontology term analysis

Gene Ontology (GO) term analysis was performed using the functional annotation tool of DAVID v6.7 (Database for Annotation, Visualization and Integrated Discovery) [44,45] for functional enrichment analysis, with the DAVID default population background for *Homo sapiens*. Analysis was performed on the individual lists, and the reported p-value was calculated using a modified Fisher Exact test (*p*-values ≤ 0.05 were considered as significant).
