# Supplementary material for: A Membrane-Type-1 Matrix Metalloproteinase (MT1-MMP) – Discoidin Domain Receptor 1 Axis Regulates Collagen-Induced Apoptosis in Breast Cancer Cells
Source: PLoS One. 2015 Mar 16;10(3):e0116006. doi: 10.1371/journal.pone.0116006 (PMC4638154; doi:10.1371/journal.pone.0116006)
Supplement: S1 Table — (DOCX) [file pone.0116006.s024.docx]

**Supplementary Table 1.** Sequences of RT-PCR primers used.

| RNA species | Primers (5’-3’) | |
| --- | --- | --- |
|  | Sense | Antisense |
| 28S rRNA | GTTCACCCACTAATAGGGAACGTGA | GATTCTGACTTAGAGGCGTTCAGT |
| ANGPTL4 | GCAGAAGCTTAAGAAGGGAATCT | GTCTTCTTCTCTGTCCACAAGTTT |
| BIK | GGACCCTATGGAGGACTTCGATT | CCTTAAGTGTGGTGAAACCGTCC |
| GJA1 | GGTCTGAGTGCCTGAACTTGCC | AAGTACTGACAGCCACACCTTCC |
| HNRNPA2B1 | GAGGTGGTTATGACAACTATGGA | CCTCTCCTATTTATACAGTGAAGC |
| HSPB8 | GGTGTCTGGCAAACATGAAGA | GAAACTGCTCTCTCCAAATGTT |
| ITGB1 | GGGGTGAATGGAACAGGGGAAAAT | GACTTTCAGGGATGCCTTCGCTTT |
| NRP1 | CACAGTGGAACAGGTGATGACTTC | AACCATATGTTGGAAACTCTGATTGT |
| TIMP3 | CTTCTGCAACTCCGACATCGTGAT | CAGCAGGTACTGGTACTTGTTGAC |
|  |  |  |
|  |  |  |
|  |  |  |
