# Supplementary material for: A Membrane-Type-1 Matrix Metalloproteinase (MT1-MMP) – Discoidin Domain Receptor 1 Axis Regulates Collagen-Induced Apoptosis in Breast Cancer Cells
Source: PLoS One. 2015 Mar 16;10(3):e0116006. doi: 10.1371/journal.pone.0116006 (PMC4638154; doi:10.1371/journal.pone.0116006)
Supplement: S5 Table — (DOCX) [file pone.0116006.s028.docx]

**Table S5.** List of genes consistently modulated by MT1-MMP in MCF-7 cells plated on 2D plastic during 24, 48 and 72 hours.

| **Genes up-regulated in MT1 cells on 2D Plastic** | | |
| --- | --- | --- |
| Probe Id | Gene symbol |  |
| ILMN_2148527 | H19 | H19, imprinted maternally expressed transcript (non-protein coding) |
| ILMN_2148527 | IGSF1 | immunoglobulin superfamily, member 1 |
| ILMN_1806603 | MESP1 | mesoderm posterior 1 homolog |
| ILMN_1707124 | TFPI | tissue factor pathway inhibitor (lipoprotein-associated coagulation inhibitor) |
| ILMN_2233314 | SPANXA1 | sperm protein associated with the nucleus, X-linked, family member A1 |
| ILMN_2211030 | SPANXB1 | sperm protein associated with the nucleus, X-linked, family member B1 |
|  |  |  |
| **Genes down-regulated in MT1 cells on 2D Plastic** | | |
| Probe Id | Gene symbol |  |
| ILMN_1799744 | GALC | galactosylceramidase |
| ILMN_2132982 | IGFBP5 | insulin-like growth factor binding protein 5 |
| ILMN_1652409 | SPATA7 | spermatogenesis associated 7 |
| ILMN_1762561 | PLA2G10 | phospholipase A2, group X |
| ILMN_1734276 | PMEPA1 | prostate transmembrane protein, androgen induced 1 |
