# Supplementary material for: A Membrane-Type-1 Matrix Metalloproteinase (MT1-MMP) – Discoidin Domain Receptor 1 Axis Regulates Collagen-Induced Apoptosis in Breast Cancer Cells
Source: PLoS One. 2015 Mar 16;10(3):e0116006. doi: 10.1371/journal.pone.0116006 (PMC4638154; doi:10.1371/journal.pone.0116006)
Supplement: S6 Table — (DOCX) [file pone.0116006.s029.docx]

**Table S6**. List of genes modulated by MT1-MMP in MCF-7 cells embedded within 3D COL1.

| 24 hours | | 48 hours | | 72 hours | |
| --- | --- | --- | --- | --- | --- |
| *Up* | *Down* | *Up* | *Down* | *Up* | *Down* |
| \| HIST1H4C \| \| --- \| \| PMEPA1 \| \| SNAR-A1 \| | \| DIO2 \| \| --- \| \| HIST3H2A \| \| HSPE1 \| \| PABPC1 \|   *FAM102A* | \| **ADM** \| \| --- \| \| **ANGPTL4** \| \| **BNIP3** \| \| **CA9** \| \| **DDIT4** \| \| **IGFBP5** \| \| **MT1X** \| \| **NDRG1** \| \| **PFKFB3** \| \| **PLIN2** \| \| **S100A4** \| \| **WISP2** \| \| ALDOC \| \| DPYSL4 \| \| EYA2 \| \| OAZ2 \| \| PFKFB4 \| | \| **BIK** \| \| --- \| \| **KLF2** \| \| **RN7SK** \| \| **RNU1-1** \| \| **RNU1-5** \| \| **RNU1-9** \| \| **RNU4-2** \| \| MGP \| \| MUCL1  *FAM102A* \| | \| **ADM** \| \| --- \| \| **ANGPTL4** \| \| **BNIP3** \| \| **CA9** \| \| **DDIT4** \| \| **IGFBP5** \| \| **MT1X** \| \| **NDRG1** \| \| **PFKFB3** \| \| **PLIN2** \| \| **S100A4** \| \| **WISP2** \| \| AHNAK \| \| BHLHE40 \| \| CAV1 \| \| CAV2 \| \| CLIC3 \| \| COL5A1 \| \| ERRFI1 \| \| FAM174B \| \| FOS \| \| H19 \| \| HIST1H4H \| \| LAMA5 \| \| LOC729978 \| \| MALL \| \| MARCKS \| \| MPZL2 \| \| PHLDA1 \| \| S100A6 \| \| TUBB3 \| \| TXNIP \| \| ZNF185 \| | \| **BIK** \| \| --- \| \| **KLF2** \| \| **RN7SK** \| \| **RNU1-1** \| \| **RNU1-5** \| \| **RNU1-9** \| \| **RNU4-2** \| \| DUSP23 \| \| RN5S9 \| \| RNU1-3 \| |

*Italic* : genes similarly modulated after 24h and 48h

**Bold** : genes similarly modulated after 48h and 72h
